# Prebiotic properties of *Bacillus coagulans* MA-13: production of galactoside hydrolyzing enzymes and characterization of the transglycosylation properties of a GH42 β-galactosidase

**DOI:** 10.1186/s12934-021-01553-y

**Published:** 2021-03-18

**Authors:** Martina Aulitto, Andrea Strazzulli, Ferdinando Sansone, Flora Cozzolino, Maria Monti, Marco Moracci, Gabriella Fiorentino, Danila Limauro, Simonetta Bartolucci, Patrizia Contursi

**Affiliations:** 1 Department of Biology, University of Naples Federico II, 80126 Naples, Italy; 2 Division of Biological Systems and Engineering, Lawrence Berkeley National Laboratory, Berkeley, CA 94720 USA; 3 Task Force On Microbiome Studies, University of Naples Federico II, Naples, Italy; 4 Department of Chemical Sciences, University of Naples Federico II, 80126 Naples, Italy; 5 CEINGE Advanced Biotechnologies, University of Naples Federico II, 80145 Naples, Italy; 6 Institute of Biosciences and BioResources—National Research Council of Italy, Naples, Italy; 7 BAT Center—Interuniversity Center for Studies On Bioinspired Agro-Environmental Technology, University of Napoli Federico II, Portici, NA Italy

**Keywords:** *Bacillus coagulans*, α-galactosidase, β-galactosidase, Transgalactosylation, Galacto-oligosaccharides, Prebiotics, Thermophilic

## Abstract

**Background:**

The spore-forming lactic acid bacterium *Bacillus coagulans* MA-13 has been isolated from canned beans manufacturing and successfully employed for the sustainable production of lactic acid from lignocellulosic biomass. Among lactic acid bacteria, *B. coagulans* strains are generally recognized as safe (GRAS) for human consumption. Low-cost microbial production of industrially valuable products such as lactic acid and various enzymes devoted to the hydrolysis of oligosaccharides and lactose, is of great importance to the food industry. Specifically, α- and β-galactosidases are attractive for their ability to hydrolyze not-digestible galactosides present in the food matrix as well as in the human gastrointestinal tract.

**Results:**

In this work we have explored the potential of *B. coagulans* MA-13 as a source of metabolites and enzymes to improve the digestibility and the nutritional value of food. A combination of mass spectrometry analysis with conventional biochemical approaches has been employed to unveil the intra- and extra- cellular glycosyl hydrolase (GH) repertoire of *B. coagulans* MA-13 under diverse growth conditions. The highest enzymatic activity was detected on β-1,4 and α-1,6-glycosidic linkages and the enzymes responsible for these activities were unambiguously identified as β-galactosidase (GH42) and α-galactosidase (GH36), respectively. Whilst the former has been found only in the cytosol, the latter is localized also extracellularly. The export of this enzyme may occur through a not yet identified secretion mechanism, since a typical signal peptide is missing in the α-galactosidase sequence. A full biochemical characterization of the recombinant β-galactosidase has been carried out and the ability of this enzyme to perform homo- and hetero-condensation reactions to produce galacto-oligosaccharides, has been demonstrated.

**Conclusions:**

Probiotics which are safe for human use and are capable of producing high levels of both α-galactosidase and β-galactosidase are of great importance to the food industry. In this work we have proven the ability of *B. coagulans* MA-13 to over-produce these two enzymes thus paving the way for its potential use in treatment of gastrointestinal diseases.

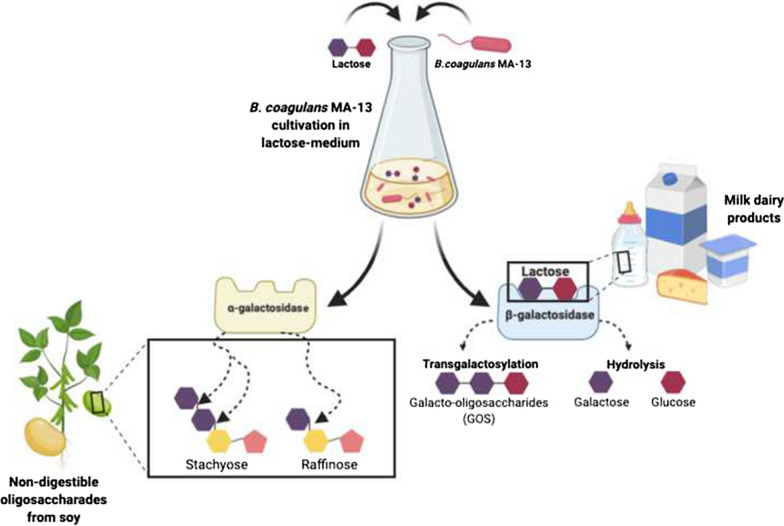

**Supplementary Information:**

The online version contains supplementary material available at 10.1186/s12934-021-01553-y.

## Background

Probiotic food production relies on the use of *Bifidobacterium*, lactic acid bacteria (LAB) as well as *Saccharomyces* species [1]. However, some *Bacillus* species have been tested as probiotics but their use is not as widespread as for traditional LAB and yeasts [2]. An attractive feature of *Bacillus spp* is the resistance to extremely harsh environments thanks to their ability to form spores and to grow under a relatively wide range of temperatures, usually up to ~ 60 °C [3, 4]. Within *Bacillus* genus*, B. coagulans* has been firstly discovered in spoiled canned milk and afterwards in other food sources. Recently, a novel thermophilic *B. coagulans* strain, designed as MA-13, has been isolated from canned beans manufacturing and shown to be able to produce lactic acid from lignocellulose biomass [5]. *B. coagulans* MA-13 turned out to be exceptionally resistant to extreme conditions, such as toxic compounds derived from the thermo-acidic treatment of lignocellulose, thus pointing to this microorganism as a good candidate as probiotic especially when harsh conditions are required for food manufacturing [5, 6].

One of the most challenging food consumption issues is how to ameliorate the digestibility of nutrients made up of complex sugars [7]*.* Indeed, the intake of foods containing some not-digestible galactosides is associated with their fermentation in the large intestine, thereby building up intestinal gas and discomfort. Among these oligosaccharides, raffinose family oligosaccharides (RFOs) (i.e. stachyose, raffinose, verbascose) are abundant in legumes and consist of α-(1,6)-D-galactose unit(s)[8], linked to sucrose. Moreover, β-(1,4)-D-galactose carbohydrates, such as lactose, are mainly present in dairy products [9].

In this context, α-galactosidases (EC 3.2.1.22) and β-galactosidases (EC 3.2.1.23) catalyze the hydrolysis of α-1,6 and β-1,4 linkages in oligo- and polysaccharides containing D-galactopyranosides, respectively. Since these enzymes are often lacking in the human intestine, it would be highly beneficial to find alternative means to deliver them into the digestive system [9]. Probiotic LAB and *Bifidobacteria*, which reside normally in the small intestine, might be used as source of digestive enzymes such as α- and β-galactosidases [10]. Previous studies have demonstrated that *Bifidobacteria* and *Lactobacilli* spp. can produce these enzymes; nevertheless, only few studies have focused on the production of both α- and β-galactosidases by the same strain [10–12]. These works have demonstrated that some probiotic microorganisms are able to express both the enzymes simultaneously when metal ions [12] or different nutrients [10, 11] were added to cultures media; however, the production of α- and β-galactosidases upon exposure to a single and inexpensive carbon source, has not yet been described. Considering the economic aspects related to large-scale enzyme production, the use of growth media containing renewable sources suitable to increase enzyme expression and reduce the cost of industrial processes, would be of great interest [13].

Among the enzymes active on not-digestible oligosaccharides, β-galactosidases are attractive not only for the hydrolysis of β-galactosyl linkages, but also for their ability to synthesize prebiotics, such as galactooligosaccharides (GOS) [14]. These are produced by transgalactosylation reactions, in which the glycosyl group of one or more D‐galactosyl units is transferred onto another mono-or oligosaccharide acceptor yielding different GOS mixtures formed by di-, tri-, tetra-, and pentasaccharides [15]. Consequently, β-galactosidase producing microbes capable of performing transgalactosylation, can be used as microbial cell factories to produce GOS molecules for the selective stimulation of the gut microbiota [12].

Thermophilic microorganisms are of general interest for both basic [16–24] and applicative research [25–28]. Among them, several thermophilic strains of *B. coagulans* have been isolated and some glycosyl hydrolytic enzymes have been characterized [29–33]. Nevertheless, comprehensive studies about the intracellular and extracellular GH enzymes spectrum of this microorganism as well as the characterisation of the transglycosylation potential of β-galactosidase enzymes are lacking. In the present work, *B. coagulans* MA-13 was explored as a cell factory for the production of enzymes with the potential to produce GOS as well as to improve the digestibility and nutritional value of foods.

## Methods

### Cultivation conditions for detection of glycosyl hydrolases activity

Aliquots from *B. coagulans* MA-13 strain stored at − 80 °C were grown under standard conditions i.e. in Luria–Bertani liquid medium at 55 °C [5]. Cells were collected through centrifugation at 3000 × *g* for 15 min and homogenized by sonication (Sonicator heat system Ultrasonic Inc.) for 10 min, alternating 30 s of pulse-on and 30 s of pulse-off. Clarification of cell extracts was obtained through centrifugation at 40,000 × *g* for 30 min at 4 °C. For analysis of the extracellular proteins, the supernatant was filtered under vacuum through 0.45 μm nylon membrane (Millipore). The filtrate (secretome) was concentrated 300-fold using an Amicon Ultrafiltration System (Millipore) with a 10 kDa cut-off nitrocellulose membrane (Millipore) at room temperature and a maximum pressure of 75 MPa. Samples were stored at 4 °C for further analysis. At least three independent biological replicates were carried out.

### Functional annotation of *B. coagulans* MA-13 Glycosyl Hydrolase enzymes

Genome draft [34] was annotated by the Rapid Annotation Subsystem Technology (RAST) and dbCAN metaserver was used to generate a family classification from CAZy database [35]. The gene list was extracted by selecting the GH(s) reported in all the 3 databases used (HMMER, DIAMOND and Hotprep).

### Screening of GH activities

Cell extract and secretome of *Bacillus coagulans* MA-13 were screened for enzymatic activities over a panel of synthetic substrates: *para*-Nitrophenyl-β-D-glucopyranoside (PNP-β-glu), *ortho*-Nitrophenyl-β-D-galactopyranoside (ONP-β-gal), *ortho*-Nitrophenyl-β-D-glucopyranoside (ONP-β-glu), *para*-Nitrophenyl-α-D-glucopyranoside (PNP-α-glu), *para*-Nitrophenyl-β-D-xylopyranoside (PNP-β-xyl), *para*-Nitrophenyl-α-D-mannopyranoside (PNP-α-man), *para*-Nitrophenyl-β-D-mannopyranoside (PNP-β-man), *para*-Nitrophenyl-β-L-fucopyranoside (PNP-β-fuc), *para*-Nitrophenyl-α-L-fucopyranoside (PNP-α-fuc), *para*-Nitrophenyl-α-L-rhamnopyranoside (PNP-α-rha), *para*-Nitrophenyl-β-D-galactopyranoside (PNP-β-gal), *para*-Nitrophenyl-α-D-galactopyranoside (PNP-α-gal), *para*-Nitrophenyl-α-L-arabinofuranoside (PNP-α-ara). Briefly, 0.8 μg of the intracellular and extracellular samples were added to the substrate (10 mM) in 100 mM citrate buffer pH 5.5 (final volume of 100 μl) and incubated in Synergy H4 Plate Reader at 55 °C. Enzymatic activity was measured by detecting the release of nitrophenol at 405 nm every 10 min up to 15 h. All the activities were expressed in International Units (U), corresponding to the quantity of enzyme(s) able to release 1 μmole of PNP-OH (millimolar extinction coefficient, 18.5 mM^−1^ cm^−1^) or ONP-OH per minute (millimolar extinction coefficient, 4.6 mM^−1^ cm^−1^). The experiments were carried out with at least three technical and biological replicates. The acceptable standard deviation was less than 20% of the mean.

Enzymatic activity on PNP-α-gal, ONP-β-gal, PNP-β-gal, PNP-α-ara, PNP-α-glu was investigated also through zymography in a 7% SDS PAGE as previously described [36]. After renaturation, the gel was incubated with 20 mM of each substrate at 55 °C for a time ranging between 10 and 60 min, until a clear halo of hydrolysis was visible on the gel. Activity bands were excised for identification of the enzyme(s) through mass spectrometry.

### Selective Growth Conditions for expression of α- and β- galactosidases

*B. coagulans* MA-13 was grown under standard conditions up to exponential growth phase (0.5 OD_600_/ml) and cells were collected through centrifugation at 3000 × *g* for 15 min. Pellets (25 OD_600nm_) were washed with milliQ water before resuspension in 50 ml of selective media. For extracellular detection of α-galactosidase, the medium contained 0.1% yeast extract (YE) and either 1% locust bean gum or diverse agri-food residues (such as rice hull). Cells were collected along with the supernatant after 24 h. Cell extracts were prepared by resuspending pellets in lysis buffer, i.e. B-PER solution (Thermo Fisher Scientific) plus lysozyme (1 mg/ml) and then incubated at 37 °C for 1 h. The lysed cells were clarified through centrifugation at 40,000 × *g* for 20 min at 4 °C. Intra- and extracellular α-galactosidase activities were analysed by testing 0.25 μg and 20 μl of supernatant on 150 μl PNP-α-gal substrate (10 mM), respectively. The assays were incubated for 10 min under standard pH and T conditions (100 mM sodium citrate 5.5 and 55 °C) and the reaction was stopped by adding 150 μl 0.5 M Na_2_CO_3_ before detection at 405 nm. As control, the supernatant was tested for β-galactosidase activity. The supernatants were analysed also through zymography on PNP-α-gal, as described above [36].

For over-expression of the native β-galactosidase, pellets (5.0 OD_600nm_) of *B. coagulans* MA-13 were resuspended in two different media, either 0.1% YE or 0.1% YE with 0.1% lactose. Cells were harvested once they reached the early stationary phase (0.8 OD_600nm_/ml) and pellets were resuspended in B-PER solution as described before. For each sample, 5 μg of intracellular proteins were tested using 10 mM ONP-β-gal following the same procedure as described above.

### Protein identification by LC–MS/MS analysis

Protein bands from SDS-PAGE corresponding to those positive in zymographic assays were excised and in situ digested with trypsin in 50 mM NH_4_HCO_3_, following treatment with 10 mM DTT (Sigma-Aldrich), for 45 min at 56 °C and then with 55 mM iodoacetamide (Sigma-Aldrich) in the dark at room temperature for 30 min for cysteines reduction and alkylation, respectively. Gel bands were then incubated with 10 ng/µl trypsin overnight at 37 °C. Peptide mixtures were extracted from the gel, and then acidified by 20% trifluoroacetic acid (Sigma-Aldrich), and dried by a Speed-Vac system (Thermo Fisher Scientific, USA). Peptide mixtures were re-suspended in 0.2% Formic Acid and analysed by nano-LC–MS/MS on a 6530 Q-TOF LC/MS equipped with a CHIP-CUBE system and coupled with a capillary 1100 HPLC system (Agilent Technologies, Santa Clara, California, USA). Each peptide sample was then fractionated with a gradient of eluent B (0,2% formic acid, 95% acetonitrile LC–MS Grade) from 5 to 75% for 100 min and eluent A (0,2% formic acid, 2% acetonitrile LC–MS Grade). Data Dependent Acquisition method was set as follows: MS scan range was from 300 to 2400 m/z; MS/MS scans from 100 to 2000 m/z were acquired for the five most abundant + 2 or + 3 charged precursor ions (top 5) in each MS scan, applying a dynamic exclusion window of 30 s.

LC–MS/MS raw data were processed and then employed for protein identification by using licensed Mascot software (Matrix Science, Boston, USA) to search in a protein-encoding genes (PEGs) database containing *B. coagulans* MA-13 predicted protein sequences. The main parameters employed for identifications were: mass tolerance value of 10 ppm for precursor ions and 0.6 Da for MS/MS fragments; trypsin as the proteolytic enzyme; missed cleavages maximum value of 1; Cys carbamidomethylation as fixed modifications; pyroglutamate (peptide N-terminal Gln) and Met oxidation as variable modifications. Candidates with at least 2 assigned peptides with an individual MASCOT score > 10 were considered significant for identification [37].

The identified proteins were compared to sequences present in a complete annotated database (UniProt) by using BLAST Search Form. Best alignments showing the minimum value for E- values were considered.

### Cloning and sequencing of the β-galactosidase gene

A single colony of *B. coagulan*s MA-13 was inoculated into LB liquid medium and genomic DNA was isolated using the LETS (lithium, EDTA, Tris, and SDS) buffer method [34, 38]. The gene (locus tag: E2E33_010705), encoding for a putative β-galactosidase, was amplified by polymerase chain reaction (PCR) using the primers 5′GAGGAATGCGTGCCATGGTAAAAAAACAT3′ (*Nco*I restriction site is underlined), 5′ATCCGGGCGCCTCGAGTTTTTCAATTAC3′ (*Xho*I restriction site is underlined) and Taq DNA Polymerase (Thermo Fisher Scientific). The amplification was performed with an initial denaturation at 95 °C for 3 min, followed by 25 cycles (95 °C for 30 s, 58 °C for 45 s and 72 °C for 75 s) and a final extension step at 72 °C for 10 min. The PCR products were checked by agarose gel electrophoresis and subsequently purified with QIAquick PCR purification kit (Qiagen Spa, Milan, Italy). Afterwards, the purified product was cloned in pCR4-TOPO-vector (TOPO TA CLONING Kit, Invitrogen) and its identity was confirmed by DNA sequencing (Eurofins Genomics). The insert then was subcloned in pET28b( +) vector (Novagen) using *NcoI*/*XhoI* restriction enzymes and T4 DNA ligase (Promega).

### Expression and purification of recombinant *Bc*GalB

The vector pET28b/*Bc*GalB containing the β-galactosidase gene was used for transforming *E. coli* Rosetta™(DE3) pLysS cells in order to express the recombinant protein bearing a C-terminus His-tag. The transformants were selected on LB agar plates containing 50 μg/ml kanamycin and 33 μg/ml chloramphenicol. A single colony was inoculated in 50 ml LB medium with antibiotics and incubated on an orbital shaker (180 rpm at 37 °C). Cells were diluted in 1 L of LB at 0.06–0.08 OD_600nm_ and once the culture reached 0.5–0.6 OD_600nm_ protein expression was induced overnight by adding 0.5 mM of IPTG. Cells were harvested by centrifugation at 4000 × *g* and resuspended in 100 mM sodium-phosphate pH 8.0 supplemented with a protease inhibitor cocktail tablet (Roche). Subsequently, the cells were disrupted by sonication (Sonicator: Heat System Ultrasonic, Inc.) for 10 min, alternating 30 s of pulse-on, and 30 s of pulse-off and the suspension was clarified by a centrifugation step at 40,000 × *g* for 30 min at 4 °C. *Bc*GalB was purified to the homogeneity by affinity chromatography on HisTrap column (1 mL, GE Healthcare) connected to an AKTA Explorer system. The column was equilibrated with 100 mM of sodium-phosphate pH 8.0 and 500 mM of sodium chloride buffer and elution was performed with a linear gradient of imidazole (0–250 mM). All the peak fractions were pooled and then dialyzed against 100 mM of sodium-phosphate pH 8.0 and 50 mM of sodium chloride (storage buffer). Protein concentration was estimated by Bradford assay using bovine serum albumin as standard. The monomeric molecular mass of *Bc*GalB was evaluated by SDS-PAGE analysis (12%) and purity degree was evaluated by staining the gel with Coomassie brilliant blue R-250.

### Molecular weight determination of *Bc*GalB

The native molecular weight of *Bc*GalB was obtained by gel-filtration chromatography connected to Mini DAWN Treos light-scattering system (Wyatt Technology) equipped with a QELS (quasi-elastic light scattering) module mass value and hydrodynamic radius (Rh) measurements. One milligram of protein (1 mg/ml) was loaded on a S200 column (16/60 GE Healthcare) with a flow-rate of 0.5 ml/min and equilibrated in 100 mM of sodium-phosphate pH 8.0, 1 mM DTT. Data were analyzed using Astra 5.3.4.14 software (Wyatt Technology).

### pH and temperature profiles of *Bc*GalB

The optimal pH value was determined by assaying 10 ng (≃ 0.04 Hydrolytic Units, U) of *Bc*GalB at 60 °C using ONP-β-gal as substrate in a pH range from 4.0 to 10.0. The following buffers (each 100 mM): sodium citrate (4.0–6.0), sodium phosphate (6.0–8.0), and glycine–NaOH (8.6–10.0) were used to prepare the different substrate mixtures containing 10 mM ONP-β-gal. The temperature dependence of *Bc*GalB activity was studied by assaying the enzyme from 30 to 90 °C in 0.1 M sodium phosphate pH 6.0 on ONP-β-gal.

Once determined the pH and temperature dependence of the enzyme, all the subsequent assays were performed using a reaction mixture containing 10 mM ONP-β-gal, 100 mM sodium citrate buffer pH 5.0 and ≃ 0.04 U of *Bc*GalB. Briefly, the substrate mix was incubated at 60 °C for 3 min, before adding the enzyme. The reaction was stopped after 3 min of incubation, by the addition of cold sodium carbonate 1.0 M. The concentration of the released ortho-nitrophenol (millimolar extinction coefficient, 4.6 mM^−1^ cm^−1^) was evaluated by measuring the absorbance of the mixture at 405 nm. The pH stability and thermal inactivation were analyzed by incubating the enzyme in sodium citrate (4.0–6.0), sodium phosphate (6.0–8.0) and at 45°, 50°, 55° and 60 °C, respectively. Aliquots of *Bc*GalB were withdrawn at regular time intervals to measure the residual activity under standard conditions.

### Effect of metal ions, chemicals, and monosaccharides on enzyme activity

To test the effect of metal ions on enzymatic activity, *Bc*GalB was dialysed in storage buffer supplemented with 10 mM EDTA for 2 h to get rid of metal ions present in the protein preparation. Afterwards, EDTA was removed through extensive dialysis in storage buffer. *Bc*GalB was incubated with metal ions for 5 min at room temperature (Mg^2+^, Ca^2+^, Zn^2+^, Mn^2+^, Co^2+^, Ni^2+^, K^+^, Li^+^, Cu^2+^, Fe^3+^, Na^+^) at 2 mM concentration and the enzymatic activity was measured under standard conditions with the addition of 2 mM of each metal ion in the mix assay. In relation to the activity of *Bc*GalB on lactose, the effect of Ca^2+^ was also evaluated in a reaction mixture containing 2 mM of Ca^2+^, 150 mM lactose and ≃0.04 U.

Furthermore, the inhibition effect of chemicals on *Bc*GalB activity was also tested. Non-ionic (Tween-20), ionic (SDS) detergents, reducing (DTT, β-mercaptoethanol), chelating (EDTA) and chaotropic (urea, guanidinium chloride) agents were added to the enzyme solution at 50 mM concentration for 5 min and residual activity was assayed under standard conditions.

Finally, the influence of monosaccharides on the enzymatic activity was studied too. IC_50_ (half maximal inhibitory concentration) was calculated by incubating the enzyme in the presence of D-xylose or D-arabinose or D-glucose or D-galactose or a mix of these two latter at different concentration values (0–100 mM), for 5 min at room temperature and assaying the enzymatic activity in the presence of the monosaccharides.

### Substrate specificity and kinetic parameters of *Bc*GalB

The hydrolytic activity of *Bc*GalB was tested on several substrates: PNP-β-glu, ONP-β-glu, PNP-α-glu, PNP-β-xyl, PNP-α-man, PNP-β-man, PNP-β-fuc, PNP-α-fuc, PNP-α-rha, ONP-β-gal, PNP-β-gal, PNP-α-gal, PNP-α-ara and D-lactose. The enzyme was incubated in presence of 10 mM of each substrate under standard assay conditions. When lactose was used, the amount of free-glucose released upon hydrolysis was determined using D-Glucose Assay Kit (GOPOD Format, Megazyme) according to the manufacturer’s protocol. One unit (U) is defined as the amount of enzyme required to release 1 μmol of glucose per min. In order to study the kinetic parameters of the enzyme, different concentration values of ONP-β-gal (0.1 to 20 mM) and lactose (0–500 mM) were tested. The Michaelis–Menten constant (K_M_) and V_max_ were calculated by non-linear regression analysis using GraphPad 9.0 Prism software.

### Analysis of the transgalactosylation activity of *Bc*GalB by Thin-Layer Chromatography (TLC)

The transgalactosylation experiments were performed to study homo- and hetero-condensation reactions under standard conditions (100 mM sodium citrate pH 5.0 and 60 °C). The final volume of all the reactions was 800 μl and contained 2.2 U (0.5 μg) of the enzyme mixed with 80 mM of ONP-β-gal or 35.0 U of *Bc*GalB (8 μg) with 160 mM lactose, respectively. For the hetero-condensation reactions, 40 mM ONP-β-gal was employed as donor and 40 mM PNP-β-glc or PNP-β-xyl as acceptors, in a final volume of 800 μl containing 2.2 U. These reaction conditions were established after preliminary tests (data not shown) in which different donor:acceptor ratios as well as of the enzyme amounts, were tested. Aliquots of reaction mixtures were collected at different time intervals (up to 18 h), and the reactions were stopped by incubation in dry ice for 5 min. Control reactions without enzyme were included in the analysis. The products were analyzed by TLC on silica gel 60 (F254, 0.25 mm) plates (Merck, Darmstadt, Germany) and separated using ethyl acetate/methanol/ddH_2_O (70:20:10 v/v) as eluent, or butanol/ethanol/ddH_2_O (50:30:20 v/v), for the detection of GOS from lactose. Aliquots corresponding to 0.2–2.0% of the total reaction mixture were loaded onto the TLC plate. For the detection of sugars, the TLC plates were soaked in a staining solution consisting of 4% of 1-naphthol in 10% sulphuric acid in ethanol followed by heating at 120 °C.

### ESI–MS Analysis of the galactooligosaccharides (GOS)

Transgalactosylation products were analyzed by direct ESI–MS procedure from reactions carried out for 18 h: all samples were diluted in 5% acetic acid and analyzed on a Q-ToF Premier (Waters, Milford, MA, USA), in positive mode, by direct injection into the ESI source at a flow of 10 µL/min. The source parameters were set as follows: capillary voltage = 3 kV and cone voltage = 42 kV. The acquisition range was set between 100 and 1000 m/z. All data were processed by using Mass Lynx 4.1 software (Waters, Milford, MA, USA).

## Results and discussion

The aim of this work was to exploit the potential of *B. coagulans* MA-13 as a source of enzymes to improve the hydrolysis of oligosaccharides that are indigestible by the human gut, and to produce prebiotics, such as galactooligosaccharides. A combination of mass spectrometry-based omics technologies with conventional biochemical approaches has been employed to investigate on the applicative potential of *B. coagulans* MA-13 in these biotechnological contexts.

### Screening and identification of the glycosyl hydrolases activities of *Bacillus coagulans* MA-13

#### Annotation of glycosyl hydrolases

Whilst a full functional annotation of *B. coagulans* MA-13 genome is under way (manuscript in preparation), herein, the annotation of the GH(s) repertoire using dbCAN2 meta server, is shown [35] (Table 1). Seventeen enzymes have been identified, among which some families (GH3, GH15, GH32, GH36, GH42, GH70, GH73) are represented by a single member, whereas all the others include diverse glycosyl hydrolases. A set of GH(s) connected to starch degradation which includes GH13 and GH65 representatives, mirrors the isolation source of *B. coagulans* MA-13, i.e. canned beans manufacturing which is particularly rich in starch [5, 39].

**Table 1:** Predicted GH in the genome of *B. coagulans* MA-13

NCBI Reference Sequence	GH family	Signal Peptide	RAST annotation
WP_195850490.1	GH3	N	β-glycosyl hydrolase
WP_019720988.1	CBM34 + GH13_20	N	Neopullulanase (EC 3.2.1.135)
WP_133536160.1	GH13_31	N	Oligo-1,6-glucosidase (EC 3.2.1.10)
WP_195850265.1	GH13_31	N	Oligo-1,6-glucosidase (EC 3.2.1.10)
WP_133536961.1	GH13_5	N	Glucan 1,4-α-maltohexaosidase (EC 3.2.1.98)
WP_195850265.1	CBM48 + GH13_9	N	1,4-α-glucan (glycogen) branching enzyme (EC 2.4.1.18)
WP_061575462.1	GH0	N	Phosphorylase b kinase regulatory subunit β
WP_133537568.1	CBM50 + GH18	N	Spore cortex-lytic enzyme, N-acetylglucosaminidase SleL
WP_133536804.1	CBM50 + GH18	N	spore peptidoglycan hydrolase (N-acetylglucosaminidase) (EC 3.2.1.-)
WP_133537667.1	GH32	N	Sucrose-6-phosphate hydrolase (EC 3.2.1.26)
WP_133537615.1	GH36	N	α-galactosidase (EC 3.2.1.22)
WP_133536219.1	GH42	N	β-galactosidase (EC 3.2.1.23)
WP_133536548.1	GH65	N	α,α-trehalose phosphorylase (2.4.1.64)
WP_133536158.1	GH65	N	Maltose phosphorylase (EC 2.4.1.8)
WP_133536578.1	GH65	N	Maltose phosphorylase (EC 2.4.1.8)
WP_133537168.1	GH70	Y (1–34)	hypothetical protein
WP_195850162.1	GH73	N	endo-β-N-acetylglucosaminidase (EC 3.2.1.96)

The automated CAZyme annotation has been carried out using dbCAN2 metaserver, integrated with HMMER, DIAMOND and Hotprep databases [35]. The presence of signal peptides in the proteins are reported as: Y = 100%, N = 0%. *GH* Glycosyl hydrolases, *CBM* carbohydrate-binding modules

Three GHs members belonging to families 18 and 73 are related to the sporulation pathway of *B. coagulans.* Few Carbohydrate-Binding Modules (CBMs) were found in association with GH13 and GH18 members. The presence of a sucrose-6-phosphate hydrolase (GH32) is in line with the capability of *B. coagulans* MA-13 to use molasses as an inexpensive sucrose-rich carbon source [6]. Finally, GH36 and GH42 members have been identified and interestingly lactic bacteria producing both α- and β-galactosidases are relevant for the food industry [10].

#### Screening of the intracellular and extracellular GH activities

To discover GH enzymatic activities, intracellular cell extracts and secretome of *B. coagulans* MA-13 were tested on a panel of artificial substrates. Cells were cultivated in LB rich medium with the purpose of detecting a baseline of activities under standard growing conditions. Cultures were collected at exponential growth phase (0.5–0.6 OD_600nm_) and 0.8 μg of total intracellular and extracellular protein preparations were assayed over the following substrates: PNP-β-glu, ONP-β-glu, PNP-α-glu, PNP-β-xyl, PNP-α-man, PNP-β-man, PNP-β-fuc, PNP-α-fuc, PNP-α-rha, ONP-β-gal, PNP-β-gal, PNP-α-gal, PNP-α-ara.

**Fig. 1:**
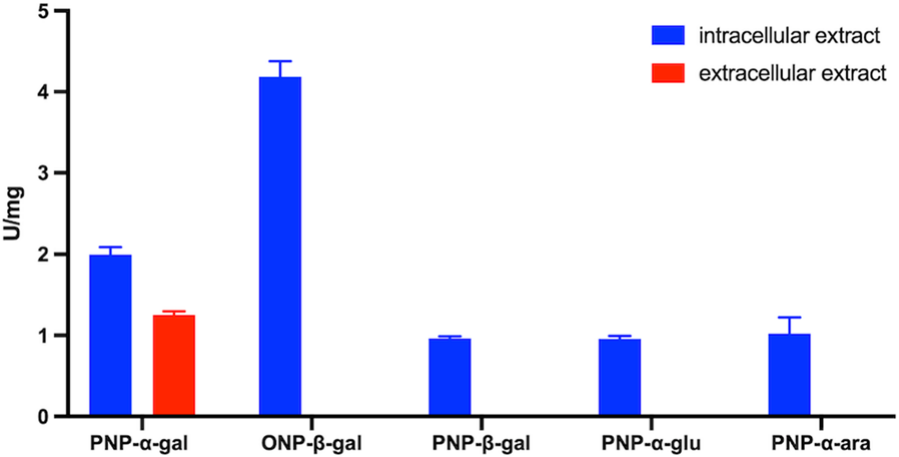
Detection of enzymatic activities on different artificial substrates

Intracellular enzymatic activities were revealed only on a subset of substrates, i.e. ONP-β-gal, PNP-β-gal, PNP-α-gal, PNP-α-glu, PNP-α-ara (Fig. 1). By comparing these findings with the annotated list of *B. coagulans* MA-13 GHs, the hydrolytic activity towards PNP-α-glu was traced back to representative(s) of the GH13 family (subfamily 31). However, the correlation with the activity on PNP-α-ara is not obvious (Table 1). The hydrolysis of β- (ONP-β-gal, PNP-β-gal) and α-galactosidic (PNP-α-gal) linkages might be linked to the GH42 and GH36 members, respectively (Fig. 1). Indeed, *B. coagulans* MA-13 genome bears two genes, i.e. locus tag: E2E33_010705 (WP_133536219.1) and locus tag: E2E33_000265 (WP_133537615.1), encoding for a GH42 and for a GH36, respectively (Table 1). As shown in Fig. 1, the specific activity recorded on ONP-β-gal and PNP-α-gal was significantly higher than on other substrates tested.

The presence of secreted GHs was verified by testing supernatants on the same substrates and the only relevant activity was detected on PNP-α-gal (Fig. 1). All together, these results indicate that enzymes hydrolyzing β- and α-galactosidic linkages represent the most relevant activities under standard growing conditions (LB medium). These enzymes catalyze the hydrolysis of terminally joined galactosidic residues in simple galactose-containing oligosaccharides as well as in complex polysaccharides and have the potential to improve the digestibility of some RFO-containing food and of milk-based products [14, 15].

#### Identification of the hydrolytic activities through mass spectrometry

To identify the enzymes involved in the hydrolysis of β-galactosidic linkages, cell extracts were analysed through zymography. Active-bands on ONP-β-gal and PNP-β-gal resided in the same upper gel region (not shown). These bands were excised, the proteins were in-gel trypsinized and the peptides were extracted and analysed by LC–ESI–MS/MS. Proteins were identified by using MASCOT search engine to explore *B. coagulans* MA-13 protein database. The sequences were compared to those present in a complete annotated database (UniProt) by using BLAST Search Form and the best alignments (minimum E-value) were obtained towards *B. coagulans* strain 36D1. As expected, α-galactosidase (Uniprot code: G2TQE8) was identified both in intra- and extracellular protein extracts together with other unrelated co-migrating proteins. The putative GH42 (Uniprot code: G2TH90) was recognized as the only enzyme potentially responsible for the hydrolytic activity on PNP-β-gal, since the other co-migrating proteins/enzymes clearly belonged to unrelated metabolic pathways (Additional file 1: Table S1). The only exception was a GH36 member (Uniprot code: G2TQE8), that, based on CAZy classification, is however not predicted to be active on PNP-β-gal substrate. Hence, the presence of this enzyme is explainable with similar migration properties to GH42 in the zymography gel. From a first inspection of proteins identified within bands active on ONP-β-gal with at least two peptides, no enzymes linked to the hydrolysis of β-1–4 linkages were found. Decreasing the detection threshold up to one peptide, a β-galactosidase (Uniprot code: G2TQE8) was detected (Additional file 1: Table S1). Overall, the results obtained from enzymatic screening and mass spectrometry analysis indicated the presence of a single enzyme (GH42, accession number: MBF8418755) involved in the hydrolysis of β-linkages. The enzyme specific activity associated to ONP-β-gal and PNP-β-gal was particularly high (Fig. 1), thus suggesting that either the enzyme was over-expressed under basal growth conditions or its specific activity was significantly high. To assess the culture conditions suitable to further increase the expression levels of β-galactosidase, the induction profile of this enzyme using a selective medium was analysed. By adding 0.1% lactose into a minimal medium (0.1% yeast), a significant increase (∼30-fold) of the β-galactosidase activity was observed (Additional file 2: Figure S1). This result is not surprising considering that most β-galactosidases play a major role in lactose metabolism and this substrate is the best carbon source to induce their maximum production in Gram + and Gram- bacteria [40, 41].

Furthermore, both cell extracts and secretome of *B. coagulans* MA-13 cells grown in LB medium were tested on PNP-α-gal, since hydrolytic activity on α-linkages was detected inside and outside the cells (Fig. 1). The activity bands of intra- and extra-cellular proteins displayed the same electrophoretic mobility, lying within the 130–180 KDa gel region. As shown by mass spectrometry analysis, the GH 36 (Uniprot code: G2TQE8) was found in both samples (Additional file 1: Table S1) suggesting that it might exert its hydrolytic activity on intracellular and extracellular α-1–6 galactans. The list of intracellular proteins identified through mass spectrometry analysis included also another GH enzyme (namely, an arabinogalactan endo-β-1,4-galactanase), however, this latter was not found in the annotated *B. coagulans* MA-13 genome (Additional file 1: Tale S1 and Table 1). The remaining co-migrating proteins identified in the extracellular and intracellular samples were related to other metabolic pathways (Additional file 1: Table S1).

The analysis of the protein sequence did not highlight any typical signal peptide (Tat or Sec system) at the N-terminus of α-galactosidase through dbCAN database (Table 1), thus raising questions on how this protein is actually secreted and why this enzyme has a dual cellular localization. A reasonable explanation is that *B. coagulans* MA-13 exploits a leader-less secretion system, namely ESAT-6 Secretion System (ESS), which has been discovered in Firmicutes and Actinobacteria [42–44]. In this system, proteins lacking a canonical signal peptide can be secreted through the combined action of two molecular components, namely EcsA and EcsB. The relative genes are both present in the *B. coagulans* MA-13 genome (Additional file 2: Figure S2) and are arranged in a cluster, likewise for other *B. coagulans* strains (not shown) and bacteria [42–44]. In some cases, a third molecular partner (EcsC) is associated to the same cluster but its role seems to be dispensable for the secretion pathway (42). Moreover, many of the proteins secreted through ESS share some distinguishing and conserved features that include a WXG amino acid motif in the central region of the protein. Interestingly, this motif has been identified in the middle of the sequence (W_368_ and G_370_) of the α-galactosidase (Accession number: MBF8416840, 730 aa) as well in the enolase (Uniprot code: G2TP79) which was found extracellularly along with the α-galactosidase (Additional file 1: Table S1).

**Fig. 2:**
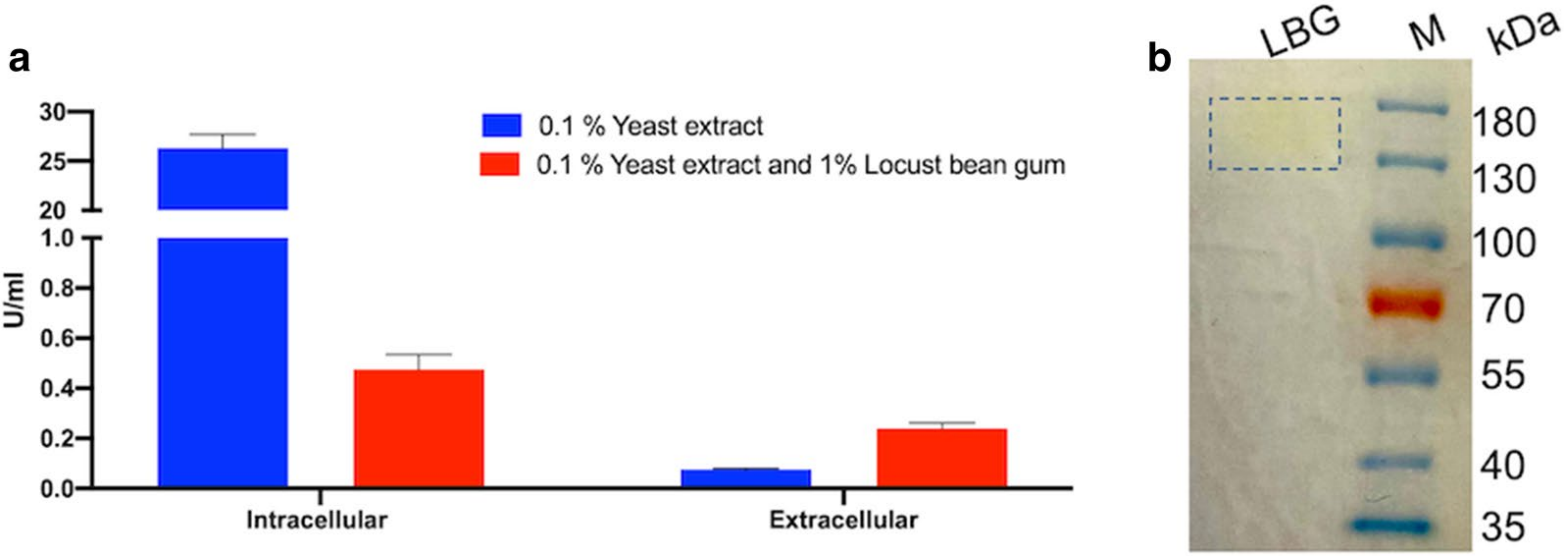
Detection of α-galactosidase activity. **a** Enzymatic assays of intracellular and extracellular extract of *B. coagulans* MA-13 on PNP-α-gal, after growth on selective medium containing locust bean gum. **b** Zymogram of supernatants from *B. coagulans* MA-13 cells grown on locust bean gum medium, using PNP-α-gal as substrate

To further confirm the presence of this enzyme in the supernatant, *B. coagulans* MA-13 was grown in a minimal medium supplemented with galactomannans (locust bean gum). These are insoluble polymers that cannot be translocated inside cells and bear α-1,6-linkages, thus being natural potential substrates of α-galactosidases. Enzymatic assays carried out on the supernatants using PNP-α-gal as a substrate, revealed that α-galactosidase was induced (about fourfold) in the presence of galactomannans compared to the control cells cultivated only in yeast (Fig. 2a). Moreover, the analysis of cell extract indicated that the levels of intracellular and extracellular enzymatic activities were similar. Conversely, the distribution of α-galactosidase was strongly biased toward its intracellular localisation when yeast was used as the only carbon source (Fig. 2a), thus suggesting that the presence in the medium of a galactose-containing polymer, such as locust bean gum, plays a role in the secretion of α-galactosidase. All together, these findings, along with the lack of a mannanase gene in *B. coagulans* MA-13 genome (Table 1), strongly supports the hypothesis that this microorganism can rely solely on the activity of an external α-galactosidase to metabolise these galactomannans. By assaying the supernatants of locust bean gum grown cells through zymography, the α-galactosidase activity was promptly revealed (Fig. 2b) and a similar result was obtained by using other complex carbon sources derived from agri-food wastes, such as rice hull (not shown). Enzymes identification by mass spectrometry was hindered by a strong contamination of polymers probably deriving from the substrates used for the detection of the in-gel activity. However, the electrophoretic mobility of this band (within the 130–180 KDa gel region) was identical to that identified as GH36 (Uniprot code: G2TQE8, Additional file 1: Table S1) thus indicating that the enzymatic activity revealed by zymography, can be ascribed to the same protein. Besides our experimental evidences, the extracellular localization of the α-galactosidase has been previously described for another closely related *B. coagulans* strain [31, 45] as well as for other soil microorganisms [46] and for *Bacillus megaterium* [47]. It is known that galactomannans are present in seeds of bean and, in general, RFOs (raffinose, stachyose, and verbascose) that contain α 1–6-linked galactose units, are particularly abundant in these legumes [9]. Since *B. coagulans* MA-13 was isolated from manufactured canned beans, the α-galactosidase, along with the β-galactosidase might be a key enzyme for the host metabolism. Indeed, manufacturing bean wastes represent a lactose-free environment. However, other genes encoding GH42 enzymes from prokaryotes are unlikely to encounter lactose, suggesting that the substrate for these enzymes in their natural environment, might also be more complex oligo- and polysaccharides [48].

The experimental evidence of the induction of β-galactosidase expression upon exposure to lactose prompted us to analyze the effect of this inexpensive substrate also on α-galactosidase production. Indeed, previous studies have reported the induction of this enzyme on galactose-containing oligosaccharides or galactose [49]. Hence, the enzymatic activity on PNP-α-gal was also measured in lactose medium and a twofold induction was observed (Additional file 2: Figure S1). However, it is not clear whether the true inducer of *B. coagulans* MA-13 α-galactosidase is lactose or galactose; indeed, the latter might be produced at high intracellular concentration as a hydrolysis product of the over-expressed β-galactosidase in the presence of lactose. Application wise, the setup of suitable growth conditions suitable for the expression of both β-and α-galactosidases is highly relevant and only a few studies have described the production of both enzymes by the same strain [11, 12, 49].

A thoroughly biochemical characterization of a closely related recombinant α-galactosidase from *B. coagulans* ATCC 7050 (identity percentage 97.4%) has been recently published [31]. Therefore, we focused on the study of the β-galactosidase enzyme, since there is no evidence about the ability of β-galactosidases from other *B. coagulans* strains to produce GOS upon transglycosylation reactions.

### Sequence analysis, cloning and expression of *Bc*GalB

The gene (E2E33_010705) encoding for the putative β-galactosidase (herein named as *Bc*GalB), has been identified within a cluster of genes encoding for a *lacI* family regulator, a hypothetical Major Facilitator Superfamily Transporter related to multi-drug resistance mechanisms and other small hypothetical proteins. This genetic arrangement is also present in *B. coagulans* ATCC 7050. The gene is, therefore, not included in an operon encoding also for a lactose-permease and a transacetylase, likewise the well-known *E. coli lac*-operon. Specifically, the hypothetical galactose-lactose permease encoding sequence is quite distant (≃7,000 nt) from the *Bc*GalB gene, thus suggesting that its expression might not be subjected to the same regulative circuit of the *lac* operon, which consists of the concomitant over-expression of the permease after exposure of cells to lactose. Accordingly, repression of *Bc*GalB in lactose-free medium, as described for *E. coli,* was not observed; rather, the enzyme was constitutively expressed under standard growth conditions and the induction fold in the presence of lactose was significant but quite low if compared to other systems (Fig. 1) [50].

**Fig. 3:**
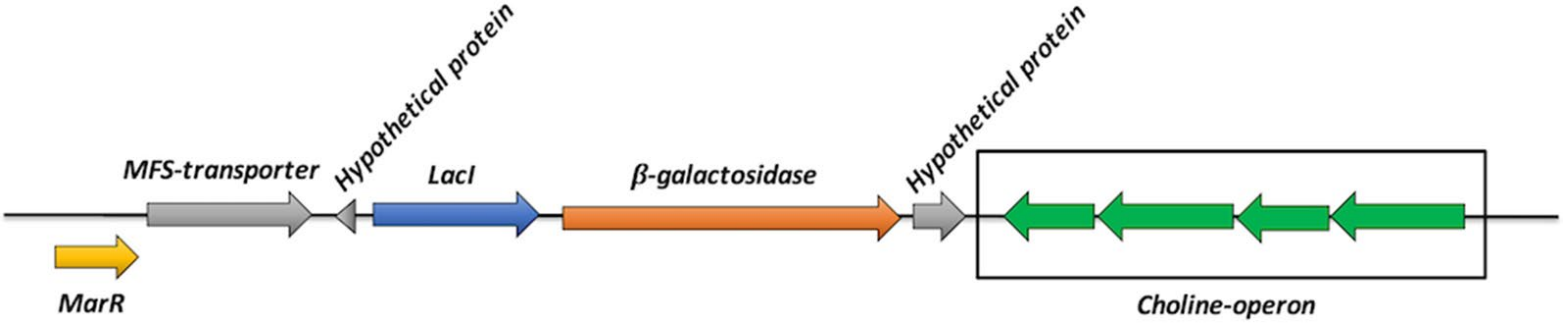
β-galactosidase genomic context in *Bacillus coagulans* MA-13

There is no report about any transcriptional cross-regulation which might account for the genetic proximity of β-galactosidase gene to choline-operon. The only functional connection has been found in a β-galactosidase from *Streptococcus mitis*, which bears a Choline Binding Domain (CBD) at its C-terminus. However, this β-galactosidase uses CBD domain as an attachment anchor to molecular components (such as lipo-teichoic acids) to bind to cell-wall. Instead, *Bc*GalB has an intracellular localization and therefore this genetic juxtaposition remains murky (Fig. 3) [51]. *Bc*GalB bears three typical domains of the GH42 family as suggested by CD-Search and other reports [33, 52]. E2E33_010705 was amplified by PCR from the genomic DNA of *B. coagulans* MA-13 and expressed in *E. coli* Rosetta™(DE3) pLysS cells as a soluble, intracellular histidine-tagged protein (C-terminus). The overexpression system and purification method applied were quite efficient, since the enzyme was purified to homogeneity by His-trap affinity chromatography, (~ 10 mg for 1 L of culture) with an yield of 82% (Additional file 3: Table S2). As revealed by the SDS-PAGE analysis (Additional file 2: Figure S3), *Bc*GalB migrated as a single band with an apparent molecular mass of ~ 75 kDa. This concurred with the molecular mass of *Bc*GalB deduced from the nucleotide sequence of the E2E33_010705 gene and the identity of the protein was verified by mass spectrometry (data not shown). The recombinant protein was analyzed by size-exclusion chromatography coupled with a triple-angle light scattering QELS. This analysis revealed that *Bc*GalB is a hexamer in solution (not shown). Since seven cysteines are present on the *Bc*GalB sequence, the enzyme was analyzed on SDS-PAGE in the presence of β-mercaptoethanol as a reducing agent (Additional file 2: Figure S3). *Bc*GalB was present only in monomeric form under this condition, thus pointing to the role of at least some of the cysteines in the oligomerization state. It is worth noting that β-galactosidases can be found in diverse oligomeric forms, such as dimeric (halophilic *Haloferax alicantei* [53]), trimeric (thermophilic *Geobacillus stearothermophilus* [54]), tetrameric (acidophilic archaeon *Sulfolobus solfataricus* [55]) and hexameric (hyperthermophilic *Thermotoga maritima* [56]) arrangements. This latter structure is uncommon among thermophilic GH42 members, whereas some GH2 β-galactosidases exhibit this supramolecular organization. To the best of our knowledge, the correlation between the hexameric structure and biochemical features of β-galactosidases is not obvious although a general correlation between oligomeric states and thermal stability has been proposed for thermophilic enzymes [36, 57].

### Characterization and stability properties of *Bc*GalB

**Fig. 4:**
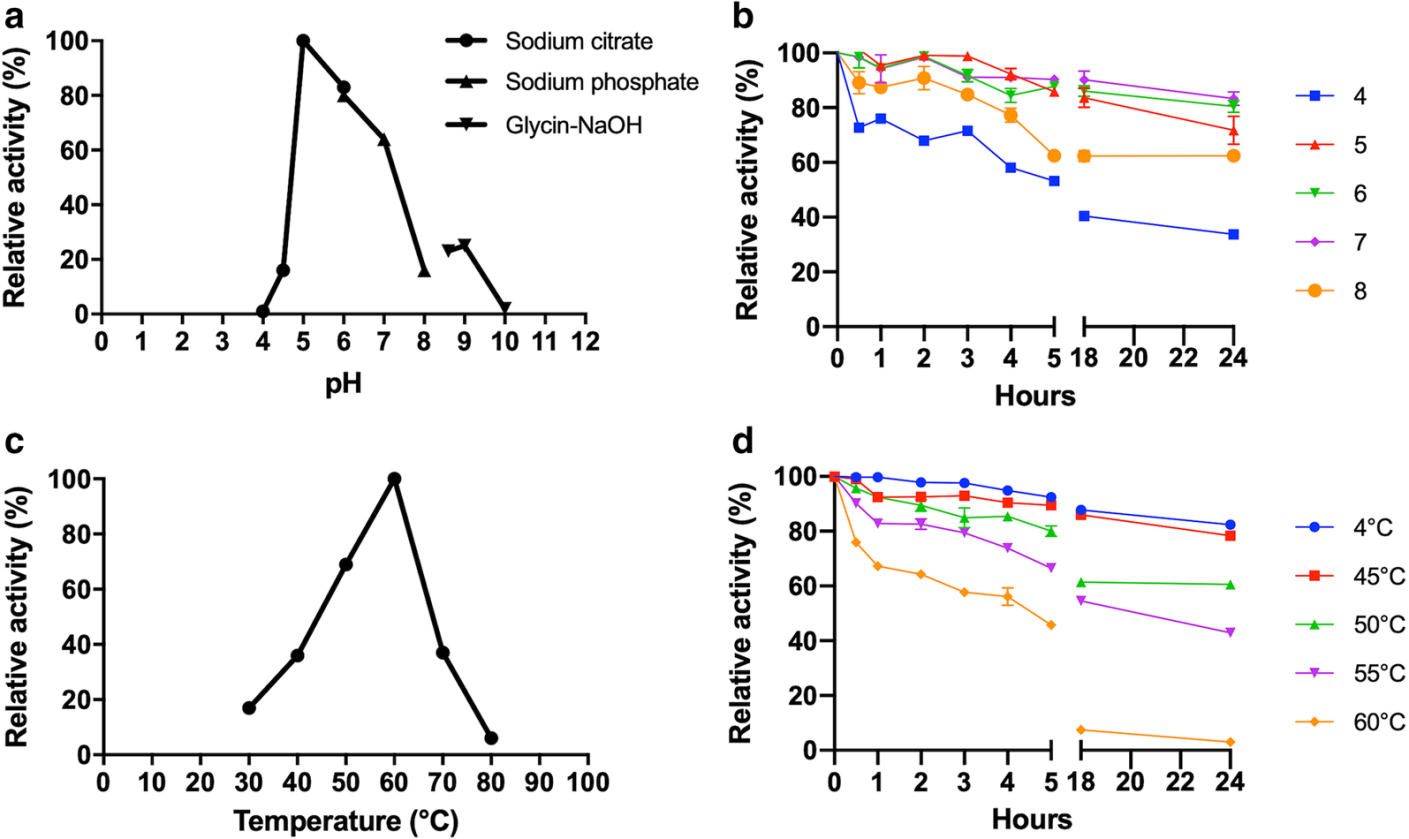
Effect of pH and temperature on the enzymatic activity. **a** The pH dependence was evaluated in different buffers ranging from pH 4.0 to pH 10.0. **b** The pH stability was studied by incubating *Bc*GalB in different buffers ranging from pH 4.0 to 8.0 up to 24 h. **c** Temperature optimum was determined by testing the enzyme in the range 30–80 °C. **d** For thermostability studies, the recombinant enzyme was incubated at different temperatures ranging from 4 to 60 °C up to 24 h

The influence of pH and temperature on the enzymatic activity was evaluated using ONP-β-gal as a substrate. After testing the enzyme in the interval 4.0–10.0, the optimal pH was set at 5.0 (Fig. 4a). Interestingly, *Bc*GalB retained 70% of its activity from 5.0 to 7.0 whilst a sharp decrease was observed at pH 4.0 (Fig. 4a). Despite this drop at acidic pH values, the enzyme exhibited a significant stability at different pH values, ranging from acidic to alkaline ones.

As shown in Fig. 4b, the enzyme retained more than 70% of its activity up to 24 h in the pH range from 5.0 to 7.0 (Additional file 2: Figure S4). The exploitation of β-galactosidases in the dairy industry is related to the optimal pH for hydrolysis [58]. Lactose is a hygroscopic sugar characterized by low solubility that causes crystallization as well as technological issues for certain products in the dairy industry. The solubility and sweetness can be increased by lactose hydrolysis into the two glucose and galactose units [59]. Hence, the feature of stability in a wide range of pH values points to *Bc*GalB as a suitable tool for slightly acid and/or sweet hydrolysis of whey.

From an industrial point of view, the enzymes should be stable both at low (preventing the proliferation of microorganisms and nutrients in milk) and at high temperatures (pasteurization) [59]. The dependence of *Bc*GalB on temperature was studied and the maximal activity was found at 60 °C (Fig. 4C), which is quite similar to that of β-galactosidases from other *B. coagulans* strains [32, 33, 60]. Moreover, *Bc*GalB exhibited high stability at a temperature of 50 °C given that approximately 60% of its initial activity was retained after incubation for up to 24 h (Fig. 4d). Moreover, the half-life at its optimal temperature was 4 h (Fig. 4d). The loss of activity at 60 °C is counterbalanced by the high specificity activity of *Bc*GalB (i.e. about 4300 U/mg, Additional file 3: Table S2) meaning that the catalytic performance of the enzyme is still consistent for an efficient hydrolysis at high temperature by employing small quantities of protein. Interestingly, the thermophilic nature and thermal stability of *Bc*GalB is exploitable for the production of lactose-free dairy products by coupling the thermization to the hydrolysis of lactose preventing microbial contamination, decreasing viscosities of the substrate solution and reducing the cost of the whole process [59].

Finally, enzymes employed in the preparation of lactose-free products are positively selected for their relatively high activity at neutral pH and stability at low temperature [59]. In this regard, the high specific activity of *Bc*GalB at neutral pH and its stability at 4 °C for up to several months match the chemical physical requirements of this biotechnological application.

### Effects of metal ions and monosaccharides on *Bc*GalB activity

It is well known that ions affect the catalytic performance of β-galactosidases. For instance, the activity of yeast enzymes isolated from *Kluyveromyces lactis* and *K. fragilis* depends on the presence of Mn^2+^ or Na^+^, and Mn^2+^, Mg^2+^, K^+^, respectively [61]. Moreover, some metal ions such as Ca^2+^, Mg^2+^ and Mn^2+^ can act as cofactors for β-galactosidases and their presence might significantly enhance their activities. Finally, it has been reported that Ca^2+^ and heavy metals inhibit the enzyme activity of several β-galactosidases. For the examination of the metal ion requirements, *Bc*GalB was assayed in the presence of 1 mM mono- and divalent ions after dialysis of the enzyme in 10 mM EDTA. Results from this study were overall in agreement with former analyses conducted on other *B. coagulans* β-galactosidases (Additional file 2: figure S5) [32, 33, 60]. Since Ca^2+^ is one of the prime elements in milk, dairy-industries processes would benefit from enzymatic activities not affected by Ca^2+^. In this regard the negligible effect of this ion up to 2 mM on the hydrolytic capability of *Bc*GalB, makes this enzyme an attractive candidate in these applications. Cu^2+^ is the only ion affecting the enzyme activity (60% reduction), as reported for other β-galactosidases. Indeed, some metal ions, such as Fe^3+^ and Cu^2+^, could inactivate the enzyme by inducing structural changes upon interaction with the protein [62, 63].

**Fig. 5:**
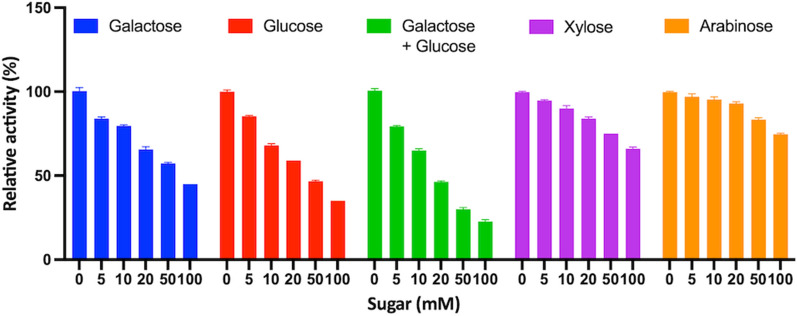
Inhibitory effect of sugars on *Bc*GalB hydrolytic activity

In order to foresee the employment of *Bc*GalB in the manufacturing of lactose-free products, the effect of galactose and glucose on enzyme activity was also studied. The inhibitory effect exerted by the lactose hydrolysis products on *Bc*GalB activity seems different from previous studies since glucose affected the *Bc*GalB enzymatic activity more than galactose (Fig. 5). Moreover, since lactose hydrolysis produces equimolar amounts of the two sugar units, we resolved to investigate the combined influence of galactose and glucose. A stronger decrease of the enzymatic activity was observed especially at high concentration of the sugars although the effect is not additive. Furthermore, xylose and arabinose were included in these experiments since the former is an acceptor of transgalactosylation reactions whereas the latter is one of the substrates of *Bc*GalB (see below). These two monosaccharides had a minor effect on the enzymatic activity compared to galactose and glucose, since *Bc*GalB retained at least 66% of the activity at the highest concentrations tested (Fig. 5). Finally, as part of the general biochemical characterisation of *Bc*GalB, the effect of surfactants (SDS and Tween 20), reducing (DTT and β-mercaptoethanol) and chaotropic (urea and guanidine chloride) agents, was studied. The enzyme activity significantly decreased only in the presence of SDS whereas it retained at least 65% of the relative activity when tested with all the other agents (Additional file 3: Table S3).

### Catalytic properties of *Bc*GalB

The hydrolytic activity of *Bc*GalB was tested on different *ortho*- or *para*-nitrophenyl synthetic glycosides as well as on natural polysaccharide substrates and specificity of the enzyme was determined by carrying out individual reactions with each of the compounds as indicated in Material and Methods section. As shown in Table 2, the highest specific activity was recorded on ONP-β-gal, whereas the enzyme performed less efficiently on *para*-substituted substrates. As shown in Fig. 1 analysis of the intracellular cell extract revealed the presence of enzyme(s) able to hydrolyse PNP-α-ara. Interestingly, a lower but still significant activity of *Bc*GalB was found on this substrate suggesting that the enzyme is endowed with an ancillary activity on PNP-β-ara. Then, the observed enzymatic activity in the cell extract can be traced back, at least in part, to *Bc*GalB (Fig. 1; Table 2). This accessory activity is surprising, since it has never been described for other thermophilic GH 42 β-galactosidases [64] and it will be a matter of further investigation. Some β-galactosidases can support the growth of environmental microorganisms from hot springs, soils and hypersaline sites where lactose is not present, but rather plant biomasses are preferential carbon and energy sources. Since *B. coagulans* MA-13 was isolated from beans processing waste, it is conceivable that *Bc*GalB may be also involved in the hydrolysis of arabino-derived oligosaccharides in vivo (Table 2).

**Table 2:** Substrate specificity of *Bc*GalB

Substrate	Specific activity (U/mg)
ONP-β-gal	4373.4 $\pm$ 77.6
PNP-β-gal	795.9 $\pm$ 3.9
PNP-α-ara	328.5 $\pm$ 11.6
PNP-β-xyl	N.D
PNP-β-glu	N.D
D-lactose	1283.0 $\pm$ 24.7

The highest activity of *Bc*GalB is toward ONP-β-gal, whilst the enzyme is not active on the arylic compounds PNP-β-xyl and PNP-β-glu. These latter two substrates are shown since they have been used in transglycosylation reactions (see below). *N.D*. not detected

Lactose, which is the natural substrate for most β-galactosidases, is translocated inside cells through specific lactose-transporters [65]. Therefore, the hydrolytic performance of *Bc*GalB on this substrate was also studied and the specific activity was found to be 1283 U/mg, which is a quite high value compared to β-galactosidases from other *B.coagulans* strains [32, 33, 60].

The kinetic parameters of *Bc*GalB were evaluated using both the preferred artificial substrate and lactose under standard reaction conditions (Table 3). Results of this analysis highlighted that *Bc*GalB showed the highest affinity towards ONP-β-gal (K_M_ = 0.72 mM) and interestingly this value is among the lowest determined so far among mesophilic and thermophilic β-galactosidases [14, 52, 66]. Moreover, even among closely related β-galactosidases from other *B. coagulans* strains, *Bc*GalB displays the highest affinity towards this substrate [32, 33, 60].

**Table 3:** Kinetic parameters of *Bc*GalB

Substrate	K_M_ (mM)	k_cat_ (s^−1^)	k_cat_/K_M_ (mM^−1^ s^−1^)
ONP-β-gal D-lactose	0.723 136.2	5466.7 1603.7	756.2 11.8

K_M_, k_cat_ and k_cat_/K_M_ values towards the natural and artificial substrates are reported. Standard deviations were lower than 2% of the calculated values

Interestingly, the enzymatic activity on lactose was not affected by Ca^2+^ and even a slight increase (114%) was recorded (data not shown). The K_M_ was found to be higher than for the artificial substrate; however, previous studies have revealed that most GH42 β-galactosidases prefer to hydrolyse chromogenic substrates while showing weaker lactose hydrolysis activity. Although GH2 β-galactosidases perform better than GH42 representatives on lactose hydrolysis, *Bc*GalB exhibits a significant specific activity toward this substrate [32, 33, 60, 67]. Accordingly, *B. coagulans* MA-13 is able to grow on lactose by over-producing *Bc*GalB (Additional file 2: figure S1), whereas several prokaryotes possessing a GH42 gene are unable to utilize this substrate [48]. This indicates that *Bc*GalB can sustain the host metabolism through hydrolysis of either lactose or more complex oligosaccharides.

### Transgalactosylation activity of *Bc*GalB

The transgalactosylation activity of *Bc*GalB was evaluated using ONP-β-gal substrate in either auto- or hetero condensation reactions, in this latter case, with different acceptors.

**Fig. 6:**
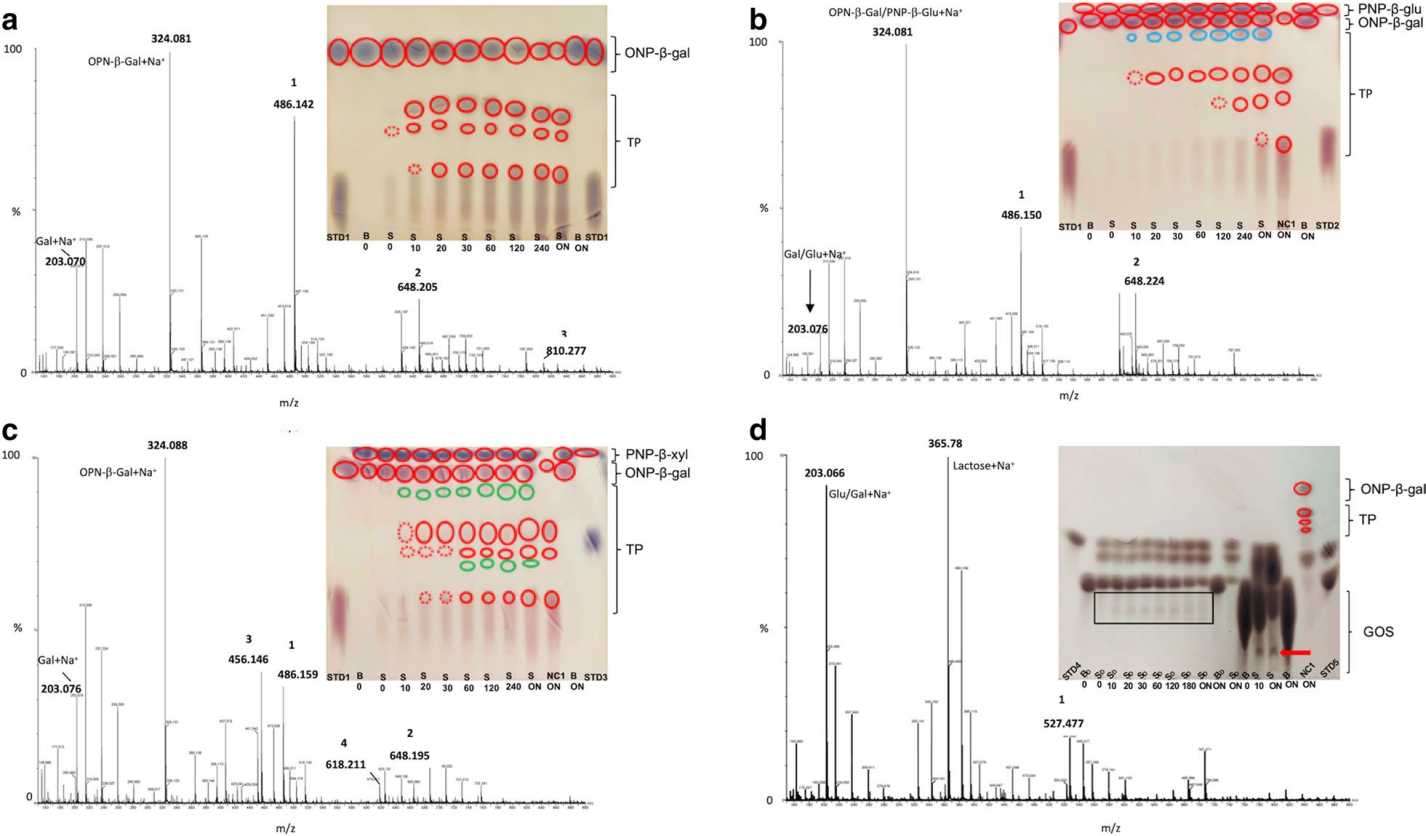
Time course of transgalactosylation reaction detected by TLC analysis. Homo-condensation reactions using ONP-β-gal as donor and acceptor (**a**). Hetero-condensation reactions performed with PNP-β-glu (**b**) and PNP-β-xyl (**c**) as acceptors and ONP-β-gal as donor. Transgalactosylation reactions using D-lactose as substrate (**d**). Red, blue and green circles show the UV signals obtained from the arylic group of ONP-β-gal, PNP-β-glu, and PNP-β-xyl respectively. *STD1*: Standard with ONP-β-gal and D-galactose, *STD2*: Standard with PNP-β-glu and D-glucose, *STD3*: Standard with PNP-β-xyl and D-xylose, *STD4*: Standard with D-glucose, *STD5*: Standard with D-galactose and D-lactose, *S*: Samples collected at different times (min), *B*: Blank at different times, *S*_*D*_: Sample diluted, *B*_*D*_: Blank diluted, *TP*: Transgalactosylation products

When ONP-β-gal was used as a donor and acceptor, TLC analysis revealed the synthesis of products of homo-galactosylation products already after 10 min of reaction (Fig. 6a, lane S 10). Moreover, additional signals were clearly visible after 20 min (Fig. 6a, lane S 20), demonstrating that in the early stages of the reaction the donor was promptly consumed in favor of the synthesis of transgalactosylation products (lower red circles).

More importantly, these compounds were not hydrolyzed by *Bc*GalB up to 18 h (Fig. 6a, lane S ON) although their complete hydrolysis was observed after the addition of fresh *Bc*GalB to the transgalactosylation mixture (data not shown). Hence, the persistence of the transgalactosylation products up to 18 h may be due to the combined effect of the partial inactivation of *Bc*GalB occurring after 4 h at 60 °C (Fig. 4d), with the inhibitory effect on the enzymatic activity due to D-galactose accumulation (Fig. 5).

*Bc*GalB is also able to synthetise hetero-oligosaccharides with PNP-β-glu and PNP-β-xyl as acceptors and ONP-β-gal as a donor. Indeed, signals that can be traced back to the formation of hetero-oligosaccharides (highlighted in blue, Fig. 6b and in green Fig. 6c), were identified togheter along with transgalactosylation products with migration properties similar to those found in homo-condensation reactions (Fig. 6b, c).

The transgalactosylation products were analysed by ESI–MS (Table 4) after carrying out all the reaction for 18 h. In all spectra, the galactose as the product of the hydrolytic activity of *Bc*GalB was detected together with the substrate(s) (ONP-β-gal, ONP-β-glu, PNP-β-xyl). For homo-condensation reactions, m/z values of 486.142, 648.205 and 810.277 corresponding to the sodium adducts of the disaccharide, trisaccharide and tetrasaccharide, were observed (Fig. 6a; Table 4). These trangalactosylation products contained galactose unit(s) (m/z = 162) and the galactose residue of ONP-β-gal (m/z = 324).

**Table 4:** Transgalactosylation products identified by ESI–MS

Acceptor:Donor	Transgalactosylation products	MNa^+^ Theoretical (Da)	MNa^+^ observed (Da)
(ONP-β-Gal: ONP-β-Gal)	1. (ONP-β-Gal + Gal) Na^+^	486.408	486.142
	2. (ONP-β-Gal + 2 Gal) Na^+^	648.565	648.205
	3. (ONP-β-Gal + 3Gal) Na^+^	810.722	810.277
(ONP-β-Gal/PNP-β-Glu: ONP-β-Gal)	1. (ONP-β-Gal/PNP-β-Glu + Gal/Glu) Na^+^	486.408	486.150
	2. (ONP-β-Gal/PNP-β-Glu + 2Gal/2Glu) Na^+^	648.565	648.224
(PNP-β-Xyl:ONP-β-Gal)	1. (ONP-β-Gal + Gal) Na^+^	486.408	486.159
	2. (ONP-β-Gal + 2Gal) Na^+^	648.565	648.195
	3. (PNP-β-Xyl + Gal) Na^+^	456.382	456.146
	4. (PNP-β-Xyl + Gal + Gal) Na^+^	618.539	618.211
(D-Lactose:D-Lactose)	1. (Lactose + Gal) Na^+^	527.477	527.202

All components were detected as adducts with Na^+^. The observed and theoretical molecular weights are reported. The ESI–MS analysis cannot distinguish between the epimer Gal and Glu, which are reported as alternatives in the interpretation of MS spectra obtained with the couple ONP-β-Gal/PNP-β-Glu: ONP-β-Gal as acceptor and donor, respectively

In the presence of glucose as acceptor, ESI–MS analysis revealed the formation of two transgalactosylation products (Fig. 6b, blue circles) counting for an increase in mass of one or two hexoses (Gal/Glu) (Fig. 6b; Table 4). These reaction products could result from both homo- and hetero-condensation of glucose and galactose molecules, although they were not distinguishable by ESI–MS analysis because of their identical molecular weight (Table 4).

In the hetero-condensation reactions containing ONP-β-Gal/PNP-β-Xyl as donor and acceptor respectively (Fig. 6c), four different products were detected by ESI–MS. Interestingly, both di-and trisaccharides deriving from homo-and hetero-condensation reactions were identified. The former contained one or two Gal molecules added to the ONP-β-Gal (m/z = 486.159 and 648.195 respectively, Table 4), whereas the latter were made up of xylose and one or two Gal units (m/z = 456.146 and 618.211 respectively, Table 4). It is noteworthy that hetero-condensation products were synthetised when either xylose or glucose were used, thus demonstrating that *Bc*GalB displays a broad acceptor specificity in transgalactosylation reactions.

Since from a biotechnological perspective, the ONP-β-gal is useless as a donor in industrial processes, the natural, plentiful and inexpensive substrate lactose was employed as the glycosyl donor and acceptor in the synthesis of glycoconjugates. A high initial lactose concentration of 160 mM was chosen to enhance GOS synthesis over hydrolysis. TLC analysis revealed the presence of hydrolysis products as well as of several GOS signals already after 10 min of incubation (Fig. 6d, lanes S_D_0 to S_D_ ON, S10 and S ON). This result indicates that *Bc*GalB is able to produce GOS at the expenses of lactose hydrolysis in a short time range. As the reaction proceeded, lactose was consumed, and glucose and galactose were formed following lactose hydrolysis. However, concurrent increase of GOS amount was not observed, as revealed by the intensity of the spots (Fig. 6d, lanes S10-S ON). This indicates that the two reactions were in a dynamic equilibrium in which GOS production reached a plateau before lactose was completely hydrolysed. ESI–MS analysis revealed a transgalactosylation product (m/z value of 527.2, Table 4) consisting of a lactose molecule increased by one galactose unit (S ON, Fig. 6d) along with the corresponding signal to the D-lactose substrate.

Collectively, these data indicate that the thermophilic *Bc*GalB is effective in the production of GOS from lactose. Moreover, lactose solubility in water is rather low in comparison to other carbohydrates; therefore, achieving a lactose concentration high enough to promote transgalactosylation reactions is a difficult task. Since lactose solubility increases exponentially with temperature, GOS synthesis can benefit from carrying out reactions with thermostable enzymes and thermophilic microorganisms.

## Conclusions

*B. coagulans* MA-13 is a versatile strain with the potential to be employed in industrial processes aimed not only at the production of value-added chemicals from lignocellulose but also of products/enzymes suitable for various industrial food applications. Indeed, this work shows the capability of this microorganism to over-produce under standard growth conditions α-and β-galactosidases that are key enzymes for improving the nutritional value of RFO- and lactose containing food. Moreover, the expression of these two enzymes can be simultaneously increased in the presence of a natural and inexpensive substrate such as lactose which is abundant in dairy wastes (i.e whey). Interestingly enough, *Bc*GalB is able to produce GOS from artificial and natural (lactose) substrates as well as to perform homo- and hetero-condensation reactions. All together these features point to *B. coagulans* MA-13 as a good candidate for the valorization of dairy waste products and for an eco-friendly and sustainable production of GOS by using whole cells.

## Supplementary Information

**Additional file 1**: **Table S1**. Intra- and extra- cellular proteins active on ONP-b-gal, PNP-b-gal and PNP-a-gal as revealed by gel zymography, were identified through LC-MS/MS method (XLSX 275 KB)

**Additional file 2**: **Figure S1**. Detection of intracellular enzymatic activities on ONP-β-gal and PNP-α-gal from *B. coagulans* MA-13 cells grown in the presence of lactose. **Figure S2**. Genetic organization of EcsA and EcsB cluster in *B. coagulans* MA-13 genome. **Figure S3**. SDS-PAGE analysis of *Bc*GalB. M. Molecular mass markers; 1 *E. coli* BL21 (DE3) Rosetta cellular extract not transformed; 2. *E. coli* BL21 (DE3) Rosetta pET28B/BcGalB cellular extract not-induced; 3. *E. coli* BL21 (DE3) Rosetta pET28B/BcGalB cellular extract induced with 0,5 mM IPTG over-night; 4. His-Trap affinity chromatography. **Figure S4**.  Relative activity of *Bc*GalB after 5, 18 and 24 hours of incubation at different pH values. **Figure S5**. Effect of metal ions on the enzymatic activity of *Bc*GalB (PPTX 1530 KB)

**Additional file 3**: **Table S2**. Purification table of *Bc*GalB. **Table S3**. Relative activity of *Bc*GalB in presence of chemicals (PPTX 43 KB)

## Data Availability

Recombinant strains described in this work are made available upon request to the corresponding author. Data sharing not applicable to this article as no datasets were generated or analysed during the current study.

## Abbreviations

LAB: Lactic acid bacteria
RFO: Raffinose family oligosaccharides
GOS: Galacto-oligosaccharides
CAZy: Carbohydrate-Active enZymes
GH: Glycoside hydrolase
g: Gravity
IPTG: Isopropyl-β-D-1-thiogalactopyranoside
ONP-β-gal: *ortho*-Nitrophenyl-β-D-galactopyranoside
PNP-α-gal: *para*-Nitrophenyl-α-D-galactopyranoside
k_cat_: Catalytic constant
U: Hydrolytic Units
K_M_: Michaelis–Menten constant
V_max_: Maximal velocity
s: Second(s)
SDS-PAGE: Sodium dodecyl sulphate–polyacrylamide gel electrophoresis
DTT: Dithiothreitol
EDTA: Ethylenediaminetetraacetic acid
TLC: Thin-Layer Chromatography
*Bc*GalB: *B. coagulans* MA-13 β-galactosidase expressed in *E.coli*
ESI–MS: Electro-Spray Mass Spectrometry
LC–MS/MS: Liquid Chromatography—Tandem Mass Spectrometry
