# Supplementary material for: Prebiotic properties of Bacillus coagulans MA-13: production of galactoside hydrolyzing enzymes and characterization of the transglycosylation properties of a GH42 β-galactosidase
Source: Microb Cell Fact. 2021 Mar 18;20:71. doi: 10.1186/s12934-021-01553-y (PMC7977261; doi:10.1186/s12934-021-01553-y)
Supplement: Supplementary file 2 — Additional file 2: Figure S1. Detection of intracellular enzymatic activities on ONP-β-gal and PNP-α-gal from B. coagulans MA-13 cells grown in the presence of lactose. Figure S2. Genetic organization of EcsA and EcsB cluster in B. coagulans MA-13 genome. Figure S3. SDS-PAGE analysis of BcGalB. M. Molecular mass markers; 1 E. coli BL21 (DE3) Rosetta cellular extract not transformed; 2. E. coli BL21 (DE3) Rosetta pET28B/BcGalB cellular extract not-induced; 3. E. coli BL21 (DE3) Rosetta pET28B/BcGalB cellular extract induced with 0,5 mM IPTG over-night; 4. His-Trap affinity chromatography. Figure S4. Relative activity of BcGalB after 5, 18 and 24 hours of incubation at different pH values. Figure S5. Effect of metal ions on the enzymatic activity of BcGalB (PPTX 1530 KB) [file 12934_2021_1553_MOESM2_ESM.pptx]

## Slide 1
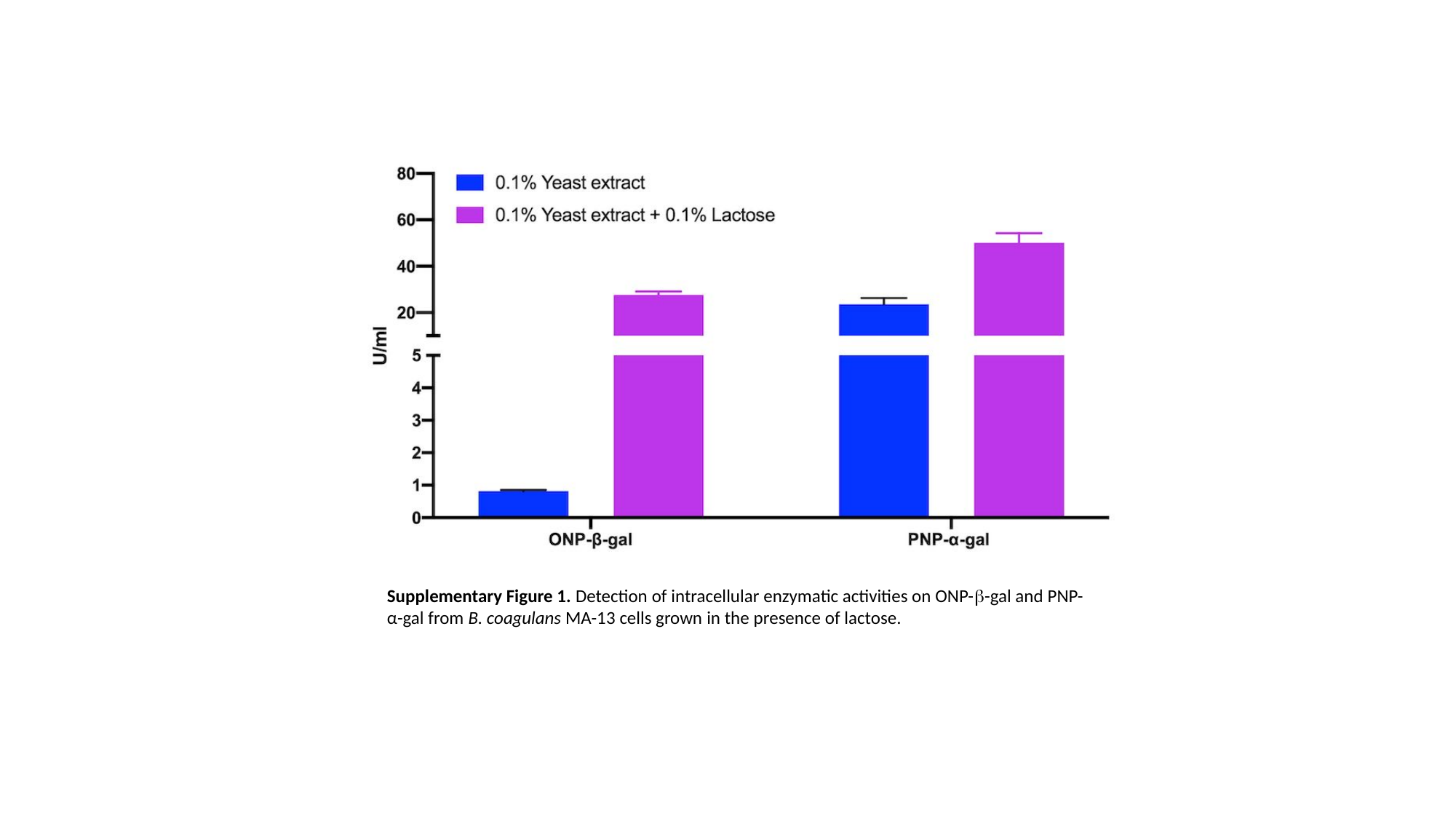

Supplementary Figure 1. Detection of intracellular enzymatic activities on ONP-b-gal and PNP-α-gal from B. coagulans MA-13 cells grown in the presence of lactose.

## Slide 2
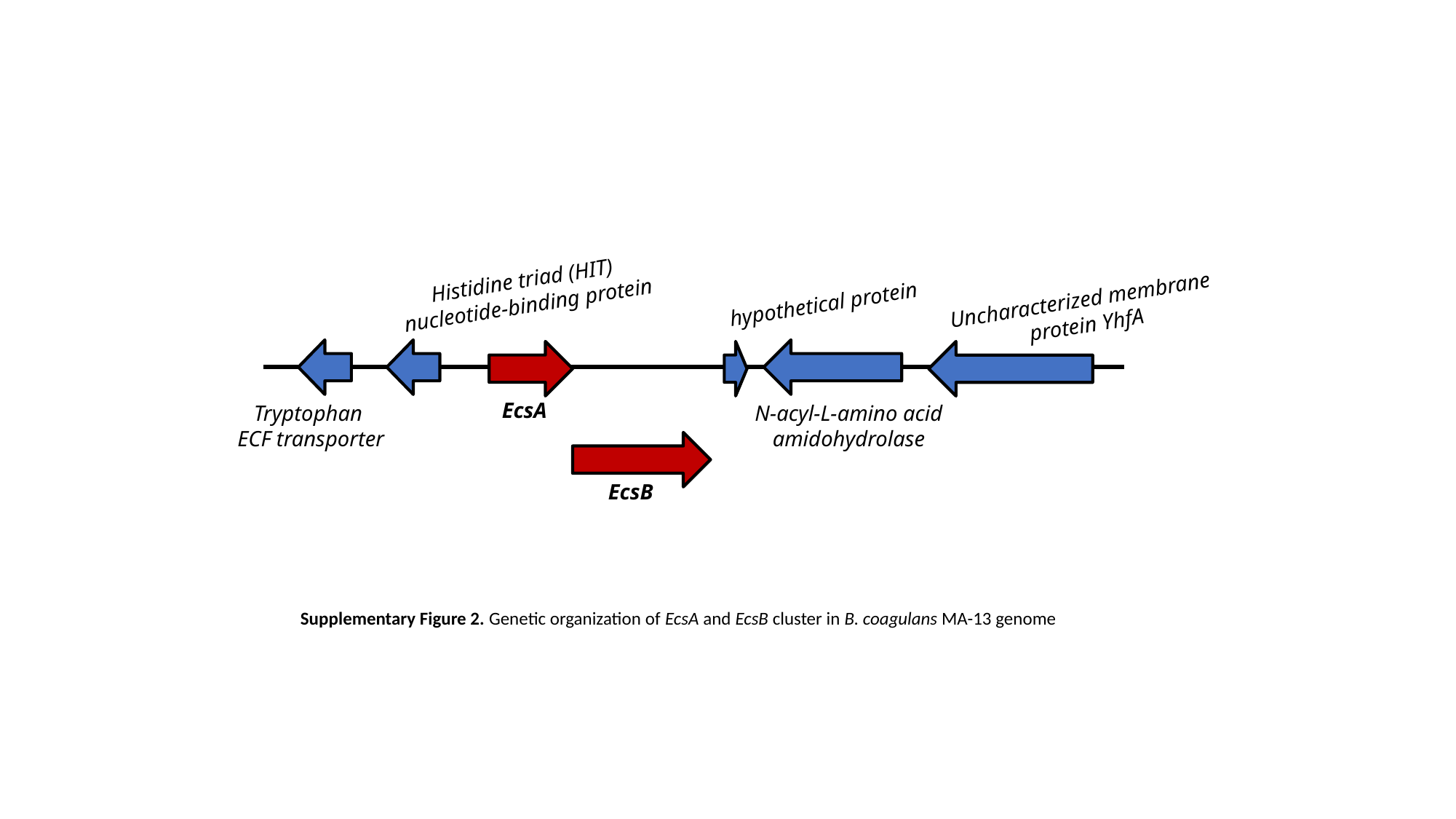

Histidine triad (HIT)
nucleotide-binding protein
Uncharacterized membrane
protein YhfA
hypothetical protein
EcsA
Tryptophan
ECF transporter
N-acyl-L-amino acid
amidohydrolase
EcsB
Supplementary Figure 2. Genetic organization of EcsA and EcsB cluster in B. coagulans MA-13 genome

## Slide 3
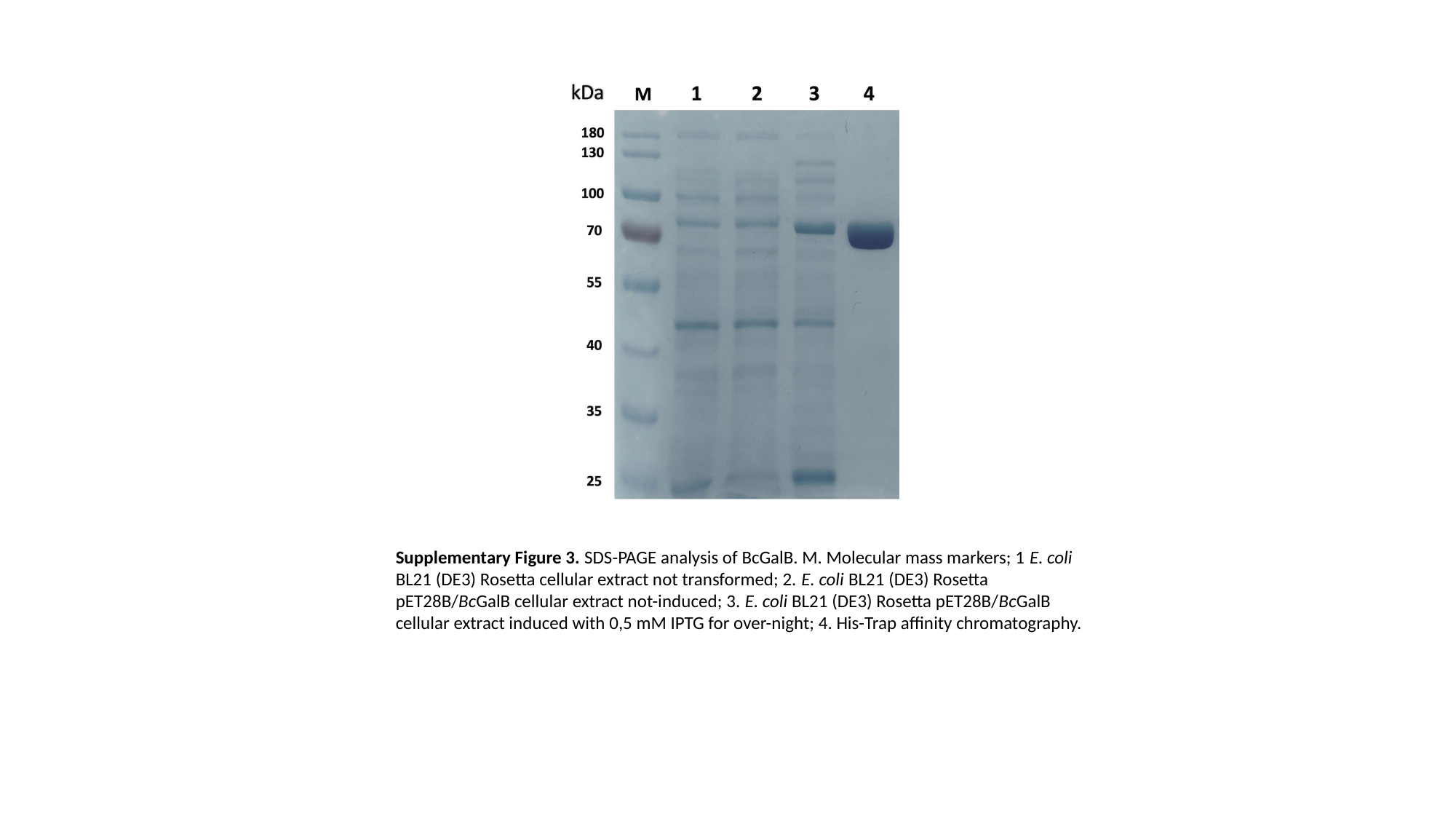

Supplementary Figure 3. SDS-PAGE analysis of BcGalB. M. Molecular mass markers; 1 E. coli BL21 (DE3) Rosetta cellular extract not transformed; 2. E. coli BL21 (DE3) Rosetta pET28B/BcGalB cellular extract not-induced; 3. E. coli BL21 (DE3) Rosetta pET28B/BcGalB cellular extract induced with 0,5 mM IPTG for over-night; 4. His-Trap affinity chromatography.

## Slide 4
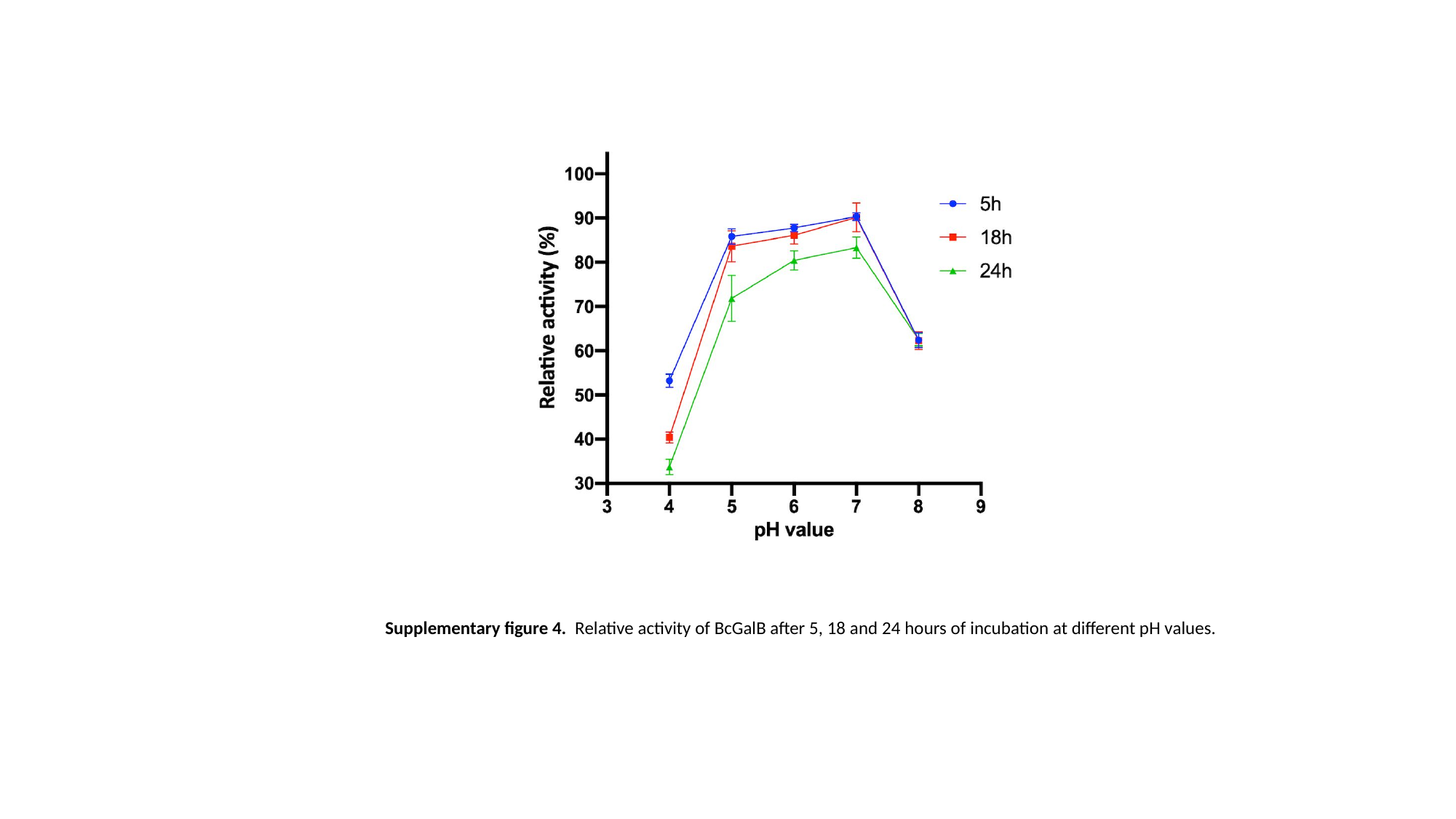

Supplementary figure 4.  Relative activity of BcGalB after 5, 18 and 24 hours of incubation at different pH values.

## Slide 5
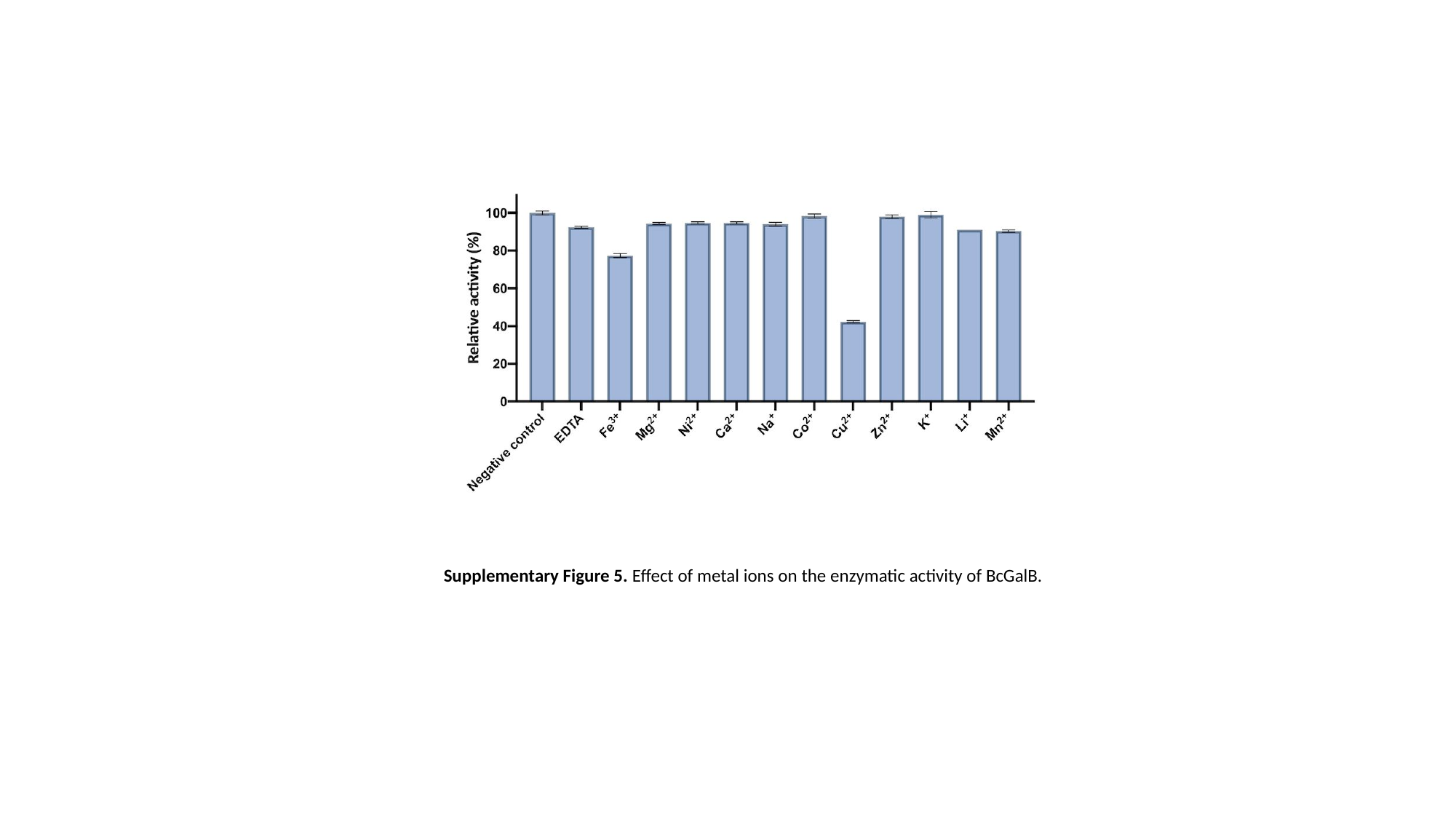

Supplementary Figure 5. Effect of metal ions on the enzymatic activity of BcGalB.
