# Supplementary material for: Prebiotic properties of Bacillus coagulans MA-13: production of galactoside hydrolyzing enzymes and characterization of the transglycosylation properties of a GH42 β-galactosidase
Source: Microb Cell Fact. 2021 Mar 18;20:71. doi: 10.1186/s12934-021-01553-y (PMC7977261; doi:10.1186/s12934-021-01553-y)
Supplement: Supplementary file 3 — Additional file 3: Table S2. Purification table of BcGalB. Table S3. Relative activity of BcGalB in presence of chemicals (PPTX 43 KB) [file 12934_2021_1553_MOESM3_ESM.pptx]

## Slide 1
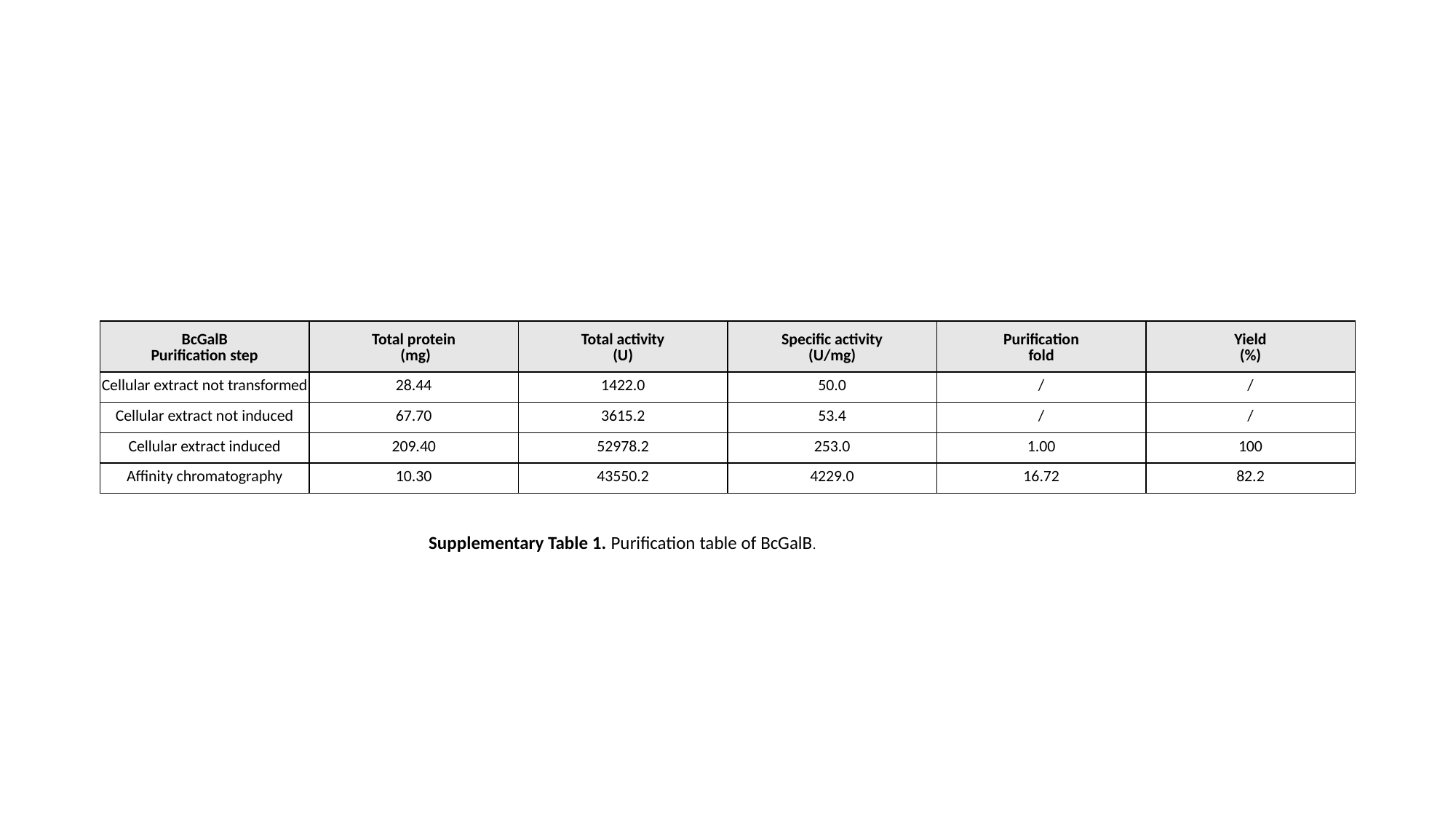

| BcGalB Purification step | Total protein  (mg) | Total activity (U) | Specific activity (U/mg) | Purification fold | Yield (%) |
| --- | --- | --- | --- | --- | --- |
| Cellular extract not transformed | 28.44 | 1422.0 | 50.0 | / | / |
| Cellular extract not induced | 67.70 | 3615.2 | 53.4 | / | / |
| Cellular extract induced | 209.40 | 52978.2 | 253.0 | 1.00 | 100 |
| Affinity chromatography | 10.30 | 43550.2 | 4229.0 | 16.72 | 82.2 |
Supplementary Table 1. Purification table of BcGalB.

## Slide 2
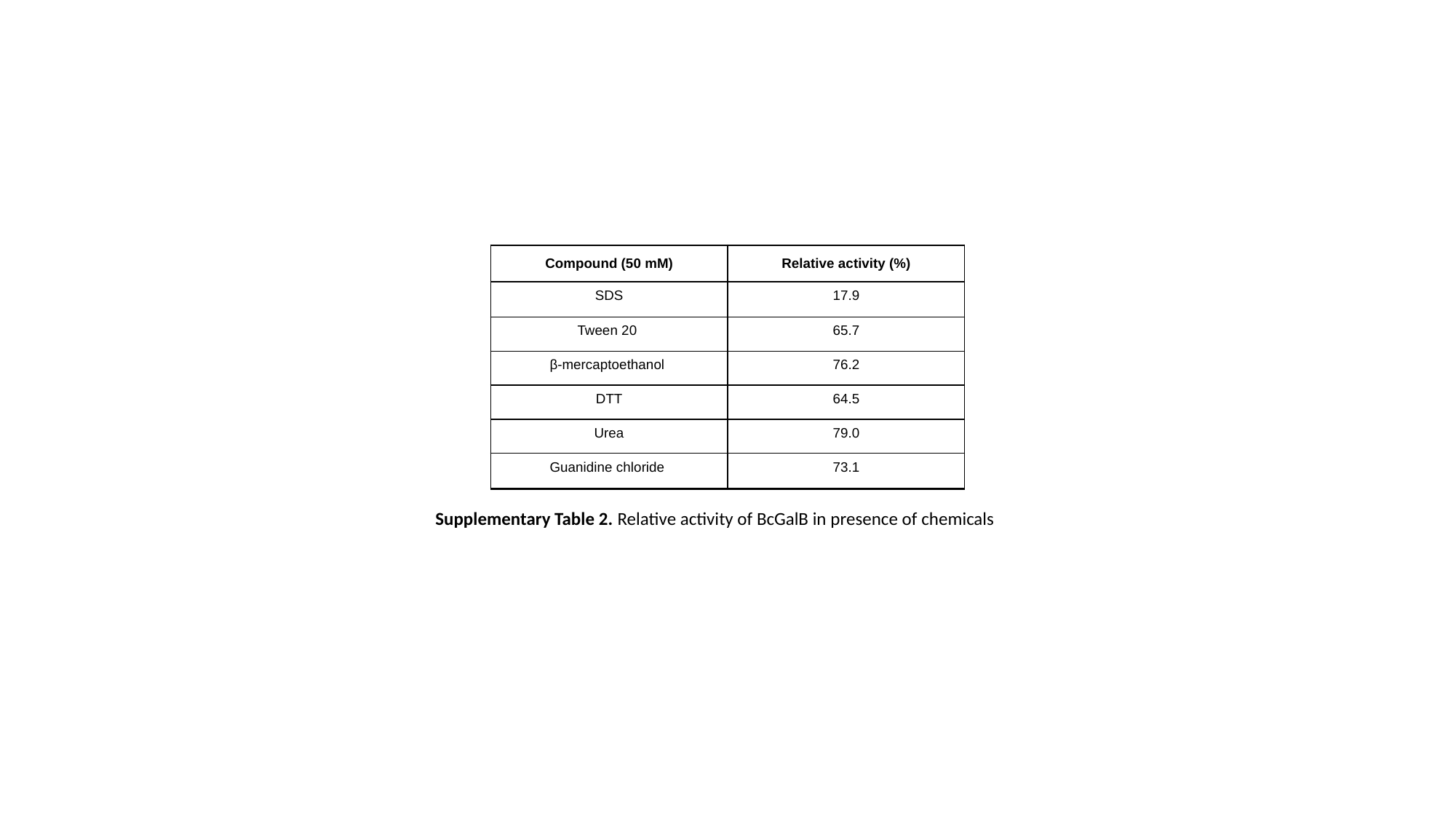

| Compound (50 mM) | Relative activity (%) |
| --- | --- |
| SDS | 17.9 |
| Tween 20 | 65.7 |
| β-mercaptoethanol | 76.2 |
| DTT | 64.5 |
| Urea | 79.0 |
| Guanidine chloride | 73.1 |
Supplementary Table 2. Relative activity of BcGalB in presence of chemicals
